# Supplementary material for: First-line targ veted therapies of advanced hepatocellular carcinoma: A Bayesian network analysis of randomized controlled trials
Source: PLoS One. 2020 Mar 5;15(3):e0229492. doi: 10.1371/journal.pone.0229492 (PMC7058293; doi:10.1371/journal.pone.0229492)
Supplement: S2 File — (DOCX) [file pone.0229492.s002.docx]

**S2 Table. Detailed Search strategy**

**Pubmed**

| #1 | randomized OR randomised OR randomly OR random |
| --- | --- |
| #2 | cancer OR carcinoma |
| #3 | liver OR hepatic OR hepatocellular |
| #4 | advanced OR unresectable OR inoperable |
| #5 | axitinib OR bevacizumab OR brivanib OR cabozantinib OR codrituzumab OR dovitinib OR erlotinib OR everolimus OR lenvatinib OR linifanib OR nintendanib OR orantinib OR ramucirumab OR regorafenib OR sorafenib OR sunitinib OR tigatuzumab OR tivantinib OR vandetanib OR targeted therapy |
| #6 | #1 AND #2 AND #3 AND #4 AND #5 |

**Embase**

| #1 | ‘randomized’/exp OR ‘randomised’/exp OR ‘randomly’/exp OR ‘random’/exp |
| --- | --- |
| #2 | ‘cancer’/exp OR ‘carcinoma’/exp |
| #3 | ‘liver’/exp OR ‘hepatic’/exp OR ‘hepatocellular’/exp |
| #4 | ‘advanced’/exp OR ‘unresectable’/exp OR ‘inoperable’/exp |
| #5 | ‘axitinib’/exp OR ‘bevacizumab’/exp OR ‘brivanib’/exp OR ‘cabozantinib’/exp OR ‘codrituzumab’/exp OR ‘dovitinib’/exp OR ‘erlotinib’/exp OR ‘everolimus’/exp OR ‘lenvatinib’/exp OR ‘linifanib’/exp OR ‘nintendanib’/exp OR ‘orantinib’exp OR ‘ramucirumab’/exp OR ‘regorafenib’/exp OR ‘sorafenib’/exp OR ‘sunitinib’/exp OR ‘tigatuzumab’/exp OR ‘tivantinib’/exp OR ‘vandetanib’exp OR ‘targeted therapy’/exp |
| #6 | #1 AND #2 AND #3 AND #4 AND #5 |

**Cochrane Library**

| #1 | randomized OR randomised OR randomly OR random |
| --- | --- |
| #2 | cancer OR carcinoma |
| #3 | liver OR hepatic OR hepatocellular |
| #4 | advanced OR unresectable OR inoperable |
| #5 | axitinib OR bevacizumab OR brivanib OR cabozantinib OR codrituzumab OR dovitinib OR erlotinib OR everolimus OR lenvatinib OR linifanib OR nintendanib OR orantinib OR ramucirumab OR regorafenib OR sorafenib OR sunitinib OR tigatuzumab OR tivantinib OR vandetanib OR targeted therapy |
| #6 | #1 AND #2 AND #3 AND #4 AND #5 in All Text |
