# Supplementary material for: First-line targ veted therapies of advanced hepatocellular carcinoma: A Bayesian network analysis of randomized controlled trials
Source: PLoS One. 2020 Mar 5;15(3):e0229492. doi: 10.1371/journal.pone.0229492 (PMC7058293; doi:10.1371/journal.pone.0229492)
Supplement: S3 File — (DOCX) [file pone.0229492.s003.docx]

Targeted drug treatment programs:

Bev+Erl: Bevacizumab 10 mg/kg intravenous injection every 14 days and erlotinib 150 mg orally daily.

Bri: Brivanib 800 mg orally once daily.

Dov: Dovitinib 500 mg orally once daily on 5 days on, 2 days off schedule.

Erl + Sor: Erlotinib 150 mg orally once a day and sorafenib 400 mg orally twice daily.

Eve + Sor: Everolimus 5 mg orally once daily and sorafenib 400 mg orally twice daily.

Len: Lenvatinib 12 mg orally once daily (for bodyweight ≥60 kg) or 8 mg once daily (for bodyweight <60 kg).

Lin: Linifanib 17.5 mg orally once daily.

Nin: Nintedanib 200 mg orally twice daily.

Ora: Orantinib 200 mg orally twice daily.

Sor: Sorafenib 400 mg orally twice daily.

Sun: Sunitinib 37.5 mg orally once daily.

Tig 2mg + Sor: Tigatuzumab 2 mg/kg orally once weekly and sorafenib 400 mg orally twice daily.

Tig 6mg + Sor: Tigatuzumab 6 mg/kg orally once weekly and sorafenib 400 mg orally twice daily.

Van 100mg: Vandetanib 100 mg orally twice daily.

Van 300mg: Vandetanib 300 mg orally twice daily.

Pla: Placebo according to the use of the experimental group to adjust.
