# Supplementary material for: First-line targ veted therapies of advanced hepatocellular carcinoma: A Bayesian network analysis of randomized controlled trials
Source: PLoS One. 2020 Mar 5;15(3):e0229492. doi: 10.1371/journal.pone.0229492 (PMC7058293; doi:10.1371/journal.pone.0229492)
Supplement: S1 Table — (DOCX) [file pone.0229492.s004.docx]

S3 Table. Risk of Bias of included studies

| Study | Random sequence generation | Allocation concealment | Blinding of participants and personnel | Blinding of outcome assessment | Incomplete outcome data | Selective reporting | Other sources of bias |
| --- | --- | --- | --- | --- | --- | --- | --- |
| Yen 2018 | Low: Computerized randomization | Low: Central allocation | High: Open-label design | Low: The endpoints were objective that could not be easily biased | Low: complete outcome data | Low: All expected outcomes had been reported | Low: Baseline features between both arms were well balanced |
| Xu 2018 | Unclear: No specific description | Low: Central allocation | High: Open-label design | Low: The endpoints were objective that could not be easily biased | Low: complete outcome data | Low: All expected outcomes had been reported | Low: Baseline features between both arms were well balanced |
| Thomas 2018 | Unclear: No specific description | Low: Central allocation | High: Open-label design | Low: The endpoints were objective that could not be easily biased | Low: complete outcome data | Low: All expected outcomes had been reported | Low: Baseline features between both arms were well balanced |
| Palmer 2018 | Low: Computerized randomization | Low: Central allocation | High: Open-label design | Low: The endpoints were objective that could not be easily biased | Low: complete outcome data | Low: All expected outcomes had been reported | Low: Baseline features between both arms were well balanced |
| Kudo Finn 2018 | Low: Computerized randomization | Low: Centralized interactive allocation | High: Open-label design | Low: The endpoints were objective that could not be easily biased | Low: complete outcome data | Low: All expected outcomes had been reported | Low: Baseline features between both arms were well balanced |
| Kudo Cheng 2018 | Low: Minimization method | Low: Centralized allocation | Low: Double-blind | Low: The endpoints were objective that could not be easily biased | Low: complete outcome data | Low: All expected outcomes had been reported | Low: Baseline features between both arms were well balanced |
| Meyer 2017 | Low: Computerized randomization | Low: Central allocation | Low: Double-blind | Low: The endpoints were objective that could not be easily biased | Low: complete outcome data | Low: All expected outcomes had been reported | Low: Baseline features between both arms were well balanced |
| Lee 2017 | Unclear: No specific description | Low: Central allocation | High: Open-label design | Low: The endpoints were objective that could not be easily biased | Low: complete outcome data | Low: All expected outcomes had been reported | Low: Baseline features between both arms were well balanced |
| Lencioni 2016 | Unclear: No specific description | Low: Central allocation | Low: Double-blind | Low: The endpoints were objective that could not be easily biased | Low: complete outcome data | Low: All expected outcomes had been reported | Low: Baseline features between both arms were well balanced |
| Koeberle 2016 | Unclear: No specific description | Low: Central allocation | High: Open-label design | Low: The endpoints were objective that could not be easily biased | Low: complete outcome data | Low: All expected outcomes had been reported | Low: Baseline features between both arms were well balanced |
| Cheng 2016 | Unclear: No specific description | Low: Central allocation | High: Open-label design | Low: The endpoints were objective that could not be easily biased | Low: complete outcome data | Low: All expected outcomes had been reported | Low: Baseline features between both arms were well balanced |
| Zhu 2015 | Low: Computerized randomization | Low: Centralized interactive allocation | Low: Double-blind | Low: The endpoints were objective that could not be easily biased | Low: complete outcome data | Low: All expected outcomes had been reported | Low: Baseline features between both arms were well balanced |
| Cheng 2015 | Low: Computerized randomization | Low: Centralized interactive allocation | High: Open-label design | Low: The endpoints were objective that could not be easily biased | Low: complete outcome data | Low: All expected outcomes had been reported | Low: Baseline features between both arms were well balanced |
| Cainap 2015 | Low: permutated-block randomization | Low: Central allocation | High: Open-label design | Low: The endpoints were objective that could not be easily biased | Low: complete outcome data | Low: All expected outcomes had been reported | Low: Baseline features between both arms were well balanced |
| Kudo 2014 | Low: Computerized randomization | Low: Central allocation | Low: Double-blind | Low: The endpoints were objective that could not be easily biased | Low: complete outcome data | Low: All expected outcomes had been reported | Low: Baseline features between both arms were well balanced |
| Johnson 2013 | Unclear: No specific description | Low: Central allocation | Low: Double-blind | Low: The endpoints were objective that could not be easily biased | Low: complete outcome data | Low: All expected outcomes had been reported | Low: Baseline features between both arms were well balanced |
| Inaba 2013 | Unclear: No specific description | Low: Centralized allocation | High: Open-label design | Low: The endpoints were objective that could not be easily biased | Low: complete outcome data | Low: All expected outcomes had been reported | Low: Baseline features between both arms were well balanced |
| Cheng 2013 | Unclear: No specific description | Low: Centralized interactive allocation | High: Open-label design | Low: The endpoints were objective that could not be easily biased | Low: complete outcome data | Low: All expected outcomes had been reported | Low: Baseline features between both arms were well balanced |
| Hsu 2012 | Low: Computerized randomization | Low: Centralized interactive allocation | Low: Double-blind | Low: The endpoints were objective that could not be easily biased | Low: complete outcome data | Low: All expected outcomes had been reported | Low: Baseline features between both arms were well balanced |
| Kudo 2014 | Unclear: No specific description | Low: Central allocation | Low: Double-blind | Low: The endpoints were objective that could not be easily biased | Low: complete outcome data | Low: All expected outcomes had been reported | Low: Baseline features between both arms were well balanced |
| Kane 2009 | Low: Computerized randomization | Low: Central allocation | Low: Double-blind | Low: The endpoints were objective that could not be easily biased | Low: complete outcome data | Low: All expected outcomes had been reported | Low: Baseline features between both arms were well balanced |
| Chen 2009 | Low: Computerized randomization | Low: Centralized interactive allocation | Low: Double-blind | Low: The endpoints were objective that could not be easily biased | Low: complete outcome data | Low: All expected outcomes had been reported | Low: Baseline features between both arms were well balanced |
