# Supplementary material for: First-line targ veted therapies of advanced hepatocellular carcinoma: A Bayesian network analysis of randomized controlled trials
Source: PLoS One. 2020 Mar 5;15(3):e0229492. doi: 10.1371/journal.pone.0229492 (PMC7058293; doi:10.1371/journal.pone.0229492)
Supplement: S2 Table — (DOCX) [file pone.0229492.s005.docx]

S8 Table. Heterogeneity and model fit.

| Endpoint | Model | standard deviation (σ) | Residual deviance | DIC statistic |
| --- | --- | --- | --- | --- |
| Time to progress | Fixed effects | NA | 11.017 | 37.230 |
|  | Random effects | 0.17 (0.03, 0.43) | 15.575 | 34.027 |
| Progression-free survival | Fixed effects | NA | 7.981 | 16.083 |
|  | Random effects | 0.18 (0.01, 043) | 8.316 | 16.683 |
| Overall survival | Fixed effects | NA | 15.011 | 42.221 |
|  | Random effects | 0.15 (0.01, 0.49) | 18.591 | 42.266 |
| Objective response rates | Fixed effects | NA | 19.425 | 65.873 |
|  | Random effects | 0.72 (0.31, 1.45) | 22.365 | 45.149 |
| Grade 3-5 adverse events | Fixed effects | NA | 20.445 | 75.359 |
|  | Random effects | 0.99 (0.42, 1.92) | 22.544 | 45.252 |

Note: NA: Not Available; DIC: deviance information criterion.
