# Supplementary material for: First-line targ veted therapies of advanced hepatocellular carcinoma: A Bayesian network analysis of randomized controlled trials
Source: PLoS One. 2020 Mar 5;15(3):e0229492. doi: 10.1371/journal.pone.0229492 (PMC7058293; doi:10.1371/journal.pone.0229492)
Supplement: S3 Table — (DOCX) [file pone.0229492.s006.docx]

**S20 Table. Inconsistency analysis of treatment effects (random effects models - 95% CrI).**

| **Comparison** | **Consistency model** | **Unrelated mean effects** |
| --- | --- | --- |
| **Time to progress** | | |
| Pla vs. Bri | 1.48 (1.04, 2.14) | 1.63 (0.94, 2.81) |
| Sor vs. Bri | 1.06 (0.75, 1.53) | 0.99 (0.59, 1.67) |
| Sor vs. Erl+Sor | 0.88 (0.54, 1.41) | 0.88 (0.52, 1.48) |
| Sor vs. Eve+Sor | 1.01 (0.57, 1.78) | 1.01 (0.54, 1.88) |
| Sor vs. Len | 1.59 (1.00, 2.53) | 1.59 (0.93, 2.67) |
| Sor vs. Lin | 1.32 (0.82, 2.12) | 1.32 (0.78, 2.22) |
| Sor vs. Nin | 0.73 (0.45, 1.19) | 0.73 (0.44, 1.22) |
| Pla vs. Ora | 1.16 (0.73, 1.84) | 1.16 (0.07, 1.95) |
| Sor vs. Pla | 0.72 (0.58, 0.89) | 0.73 (0.57, 0.93) |
| Sun vs. Sor | 1.26 (0.91, 1.90) | 1.26 (0.89, 1.96) |
| Tig 2mg vs. Sor | 1.12 (0.64, 1.95) | 1.12 (0.62, 2.02) |
| Tig 6mg vs. Sor | 1.15 (0.68, 1.98) | 1.15 (0.64, 2.03) |
| **Progression-free survival** | | |
| Sor vs. Len | 1.51 (0.90, 2.55) | 1.52 (0.88, 2.55) |
| Sor vs. Lin | 1.23 (0.72, 2.09) | 1.24 (0.72, 2.10) |
| Sor vs. Nin | 0.89 (0.55, 1.46) | 0.89 (0.55, 1.45) |
| Pla vs. Ora | 1.43 (0.75, 2.74) | 1.43 (0.75, 2.73) |
| Sor vs. Pla | 0.99 (0.57, 1.75) | 0.99 (0.56, 1.73) |
| Van 100mg vs. Pla | 0.64 (0.27, 1.53) | 0.64 (0.33, 1.26) |
| Van 300mg vs. Pla | 0.72 (0.30, 1.66) | 0.72 (0.38, 1.36) |
| Sun vs. Sor | 1.43 (0.75, 2.69) | 1.43 (0.76, 2.69) |
| **Overall survival** | | |
| Sor vs. Bev+Erl | 1.08 (0.57, 2.03) | 1.09 (0.53, 2.21) |
| Pla vs. Bri | 1.23 (0.83, 1.75) | 1.12 (0.60, 2.09) |
| Sor vs. Bri | 0.90 (0.61, 1.25) | 0.93 (0.53, 1.65) |
| Sor vs. Dov | 0.79 (0.46, 1.37) | 0.79 (0.42, 1.50) |
| Sor vs. Erl+Sor | 1.08 (0.67, 1.73) | 1.08 (0.60, 1.93) |
| Sor vs. Eve+Sor | 0.91 (0.51, 1.61) | 0.90 (0.47, 1.73) |
| Sor vs. Len | 1.09 (0.68, 1.72) | 1.09 (0.61, 1.94) |
| Sor vs. Lin | 0.95 (0.60, 1.52) | 0.95 (0.53, 1.68) |
| Sor vs. Nin | 1.10 (0.69, 1.74) | 1.09 (0.66, 1.83) |
| Pla vs. Ora | 0.93 (0.65, 1.32) | 0.93 (0.61, 1.41) |
| Sor vs. Pla | 0.73 (0.57, 0.92) | 0.71 (0.53, 0.95) |
| Van 100mg vs. Pla | 0.44 (0.23, 0.84) | 0.44 (0.22, 0.91) |
| Van 300mg vs. Pla | 0.60 (0.31, 1.16) | 0.60 (0.29, 1.23) |
| Sun vs. Sor | 1.30 (0.81, 2.05) | 1.30 (0.73, 2.29) |
| Tig 2mg vs. Sor | 1.24 (0.72, 2.14) | 1.25 (0.66, 2.33) |
| Tig 6mg vs. Sor | 0.84 (0.49, 1.45) | 0.85 (0.44, 1.58) |
| **Objective response rates** | | |
| Pla vs. Bri | 1.35 (0.36, 4.97) | 0.78 (0.20, 2.92) |
| Sor vs. Bri | 2.72 (0.76, 10.54) | 4.83 (1.25, 18.8) |
| Sor vs. Dov | 1.93 (0.25, 15.29) | 1.93 (0.37, 10.73) |
| Sor vs. Erl+Sor | 0.56 (0.09, 3.53) | 0.57 (0.14, 2.34) |
| Sor vs. Len | 0.32 (0.06, 1.90) | 0.32 (0.09, 1.19) |
| Sor vs. Lin | 0.58 (0.10, 3.38) | 0.58 (0.15, 2.17) |
| Sor vs. Pla | 2.02 (0.88, 5.08) | 1.47 (0.82, 3.62) |
| Sun vs. Sor | 0.42 (0.06, 2.76) | 0.41 (0.09, 1.79) |
| **Grade 3-5 adverse events** | | |
| Pla vs. Bri | 0.60 (0.09, 3.66) | 0.34 (0.04, 2.75) |
| Sor vs. Bri | 3.98 (0.62, 25.71) | 7.08 (0.86, 59.15) |
| Sor vs. Dov | 0.69 (0.06, 7.67) | 0.69 (0.08, 6.16) |
| Sor vs. Erl+Sor | 0.74 (0.07, 8.14) | 0.74 (0.09, 6.5) |
| Sor vs. Eve+Sor | 0.74 (0.07, 7.98) | 0.75 (0.09, 6.48) |
| Sor vs. Lin | 0.53 (0.05, 5.87) | 0.53 (0.06, 4.36) |
| Sor vs. Nin | 4.82 (0.77, 31.28) | 4.7 (0.9, 25.82) |
| Sor vs. Pla | 6.63 (1.45, 33.65) | 3.99 (1.09, 25.66) |
| Van 100mg vs. Pla | 0.05 (0.00, 0.95) | 0.34 (0.03, 3.46) |
| Van 300mg vs. Pla | 0.07 (0.00, 1.44) | 0.49 (0.04, 5.24) |
