# Supplementary material for: First-line targ veted therapies of advanced hepatocellular carcinoma: A Bayesian network analysis of randomized controlled trials
Source: PLoS One. 2020 Mar 5;15(3):e0229492. doi: 10.1371/journal.pone.0229492 (PMC7058293; doi:10.1371/journal.pone.0229492)
Supplement: S4 Table — (DOCX) [file pone.0229492.s007.docx]

S22 Table. Direct, Indirect, and NMA Estimates for OS with the GRADE Assessment.

| Treatment comparison | *I^2^* | Direct estimate;  HR (95% CI) | Quality of evidence | Indirect estimate;  HR (95% CI) | Quality of evidence | Network estimate;  HR (95% CrI) | Quality of evidence |
| --- | --- | --- | --- | --- | --- | --- | --- |
| Bev+Erl vs. Bri | NA | NA | NA | 0.86 (0.53, 1.40) | Low^4^ | 0.82 (0.39, 1.67) | Low^4^ |
| Bev+Erl vs. Dov | NA | NA | NA | 0.72 (0.40, 1.30) | Low^4^ | 0.73 (0.32, 1.68) | Low^4^ |
| Bev+Erl vs. Erl+Sor | NA | NA | NA | 0.99 (0.60, 1.63) | Low^4^ | 0.99 (0.46, 2.15) | Low^4^ |
| Bev+Erl vs. Eve+Sor | NA | NA | NA | 0.83 (0.45, 1.53) | Low^4^ | 0.84 (0.36, 1.95) | Low^4^ |
| Bev+Erl vs. Len | NA | NA | NA | 1.00 (0.61, 1.63) | Low^4^ | 1.00 (0.46, 2.18) | Low^4^ |
| Bev+Erl vs. Lin | NA | NA | NA | 0.88 (0.54, 1.44) | Low^4^ | 0.88 (0.40, 1.91) | Low^4^ |
| Bev+Erl vs. Nin | NA | NA | NA | 1.01 (0.56, 1.82) | Low^4^ | 1.01 (0.47, 2.21) | Low^4^ |
| Bev+Erl vs. Ora | NA | NA | NA | 0.61 (0.35, 1.08) | Very Low^4,6^ | 0.63 (0.29, 1.32) | Very Low^4,6^ |
| Bev+Erl vs. Pla | NA | NA | NA | 0.66 (0.38, 1.14) | Low^4^ | 0.67 (0.34, 1.31) | Low^4^ |
| Bev+Erl vs. Sor | NA | 0.92 (0.58, 1.48) | Low^1,4^ | NA | NA | 0.92 (0.49, 1.74) | Low^4^ |
| Bev+Erl vs. Sun | NA | NA | NA | 1.10 (0.57 2.12) | Low^4^ | 0.71 (0.33, 1.55) | Low^4^ |
| Bev+Erl vs. Tig 2mg + Sor | NA | NA | NA | 0.74 (0.38, 1.43) | Low^4^ | 0.74 (0.33, 1.73) | Low^4^ |
| Bev+Erl vs. Tig 6mg + Sor | NA | NA | NA | 1.10 (0.57, 2.12) | Low^4^ | 1.10 (0.48, 2.55) | Low^4^ |
| Bev+Erl vs. Van 100mg | NA | NA | NA | 1.50 (0.63, 3.61) | Very Low^4,6^ | 1.53 (0.59, 3.90) | Very Low^4,6^ |
| Bev+Erl vs. Van 300mg | NA | NA | NA | 1.11 (0.46, 2.66) | Very Low^4,6^ | 1.12 (0.43, 2.89) | Very Low^4,6^ |
| Bri vs. Dov | NA | NA | NA | 0.84 (0.58, 1.23) | Low^4^ | 0.88 (0.47, 1.73) | Low^4^ |
| Bri vs. Erl+Sor | NA | NA | NA | 1.15 (0.92, 1.44) | Moderate^4^ | 1.20 (0.68, 2.23) | Moderate^4^ |
| Bri vs. Eve+Sor | NA | NA | NA | 0.96 (0.64, 1.46) | Low^4^ | 1.01 (0.53, 2.02) | Low^4^ |
| Bri vs. Len | NA | NA | NA | 1.16 (0.96, 1.41) | Low^4^ | 1.21 (0.69, 2.22) | Low^4^ |
| Bri vs. Lin | NA | NA | NA | 1.02 (0.83, 1.25) | Low^4^ | 1.06 (0.60, 1.97) | Low^4^ |
| Bri vs. Nin | NA | NA | NA | 0.99 (0.80, 1.22) | Low^4^ | 1.23 (0.70, 2.24) | Low^4^ |
| Bri vs. Ora | NA | NA | NA | 0.71 (0.50, 1.01) | Very Low^4,6^ | 0.76 (0.46, 1.28) | Very Low^4,6^ |
| Bri vs. Pla | NA | 0.90 (0.65, 1.23) | Moderate^4^ | 0.77 (0.38, 1.43) | Moderate^4^ | 0.82 (0.57, 1.21) | Moderate^4^ |
| Bri vs. Sor | NA | 1.07 (0.94, 1.23) | Moderate^4^ | 1.27 (0.63, 2.56) | Moderate^4^ | 1.11 (0.80, 1.64) | Moderate^4^ |
| Bri vs. Sun | NA | NA | NA | 0.82 (0.68, 1.00) | Low^4^ | 0.86 (0.49, 1.61) | Low^4^ |
| Bri vs. Tig 2mg + Sor | NA | NA | NA | 0.86 (0.53, 1.40) | Low^4^ | 0.90 (0.48, 1.77) | Low^4^ |
| Bri vs. Tig 6mg + Sor | NA | NA | NA | 1.27 (0.79, 2.06) | Low^4^ | 1.33 (0.70, 2.62) | Low^4^ |
| Bri vs. Van 100mg | NA | NA | NA | 2.04 (0.96, 4.36) | Moderate^4^ | 1.86 (0.89, 3.92) | Moderate^4,^ |
| Bri vs. Van 300mg | NA | NA | NA | 1.50 (0.70, 3.21) | Moderate^4^ | 1.36 (0.65, 2.91) | Moderate^4^ |
| Dov vs. Erl+Sor | NA | NA | NA | 1.37 (0.92, 2.03) | Low^4^ | 1.37 (0.66, 2.81) | Low^4^ |
| Dov vs. Eve+Sor | NA | NA | NA | 1.14 (0.77, 1.94) | Low^4^ | 1.15 (0.52, 2.53) | Low^4^ |
| Dov vs. Len | NA | NA | NA | 1.38 (0.94, 2.02) | Low^4^ | 1.38 (0.68, 2.85) | Low^4^ |
| Dov vs. Lin | NA | NA | NA | 1.21 (0.82, 1.78) | Low^4^ | 1.21 (0.59, 2.47) | Low^4^ |
| Dov vs. Nin | NA | NA | NA | 1.40 (0.85, 2.30) | Low^4^ | 1.39 (0.68, 2.84) | Low^4^ |
| Dov vs. Ora | NA | NA | NA | 0.85 (0.52, 1.37) | Very Low^4,6^ | 0.86 (0.43, 1.72) | Very Low^4,6^ |
| Dov vs. Pla | NA | NA | NA | 0.91 (0.58, 1.44) | Low^4^ | 0.93 (0.51, 1.67) | Low^4^ |
| Dov vs. Sor | NA | 1.27 (0.89, 1.81) | Low^1,4^ | NA | NA | 1.27 (0.73, 2.20) | Low^4^ |
| Dov vs. Sun | NA | NA | NA | 0.98 (0.67, 1.43) | Low^4^ | 0.98 (0.48, 2.02) | Low^4^ |
| Dov vs. Tig 2mg + Sor | NA | NA | NA | 1.02 (0.56, 1.83) | Low^4^ | 1.02 (0.47, 2.20) | Low^4^ |
| Dov vs. Tig 6mg + Sor | NA | NA | NA | 1.51 (0.84, 2.71) | Low^4^ | 1.51 (0.70, 3.27) | Low^4^ |
| Dov vs. Van 100mg | NA | NA | NA | 2.07 (0.91, 4.72) | Very Low^4,6^ | 2.11 (0.86, 5.04) | Very Low^4,6^ |
| Dov vs. Van 300mg | NA | NA | NA | 1.53 (0.67, 3.48) | Very Low^4,6^ | 1.55 (0.63, 3.71) | Very Low^4,6^ |
| Erl+Sor vs. Eve+Sor | NA | NA | NA | 0.84 (0.55, 1.28) | Low^4^ | 0.84 (0.41, 1.75) | Low^4^ |
| Erl+Sor vs. Len | NA | NA | NA | 1.01 (0.81, 1.27) | Low^4^ | 1.01 (0.52, 1.97) | Low^4^ |
| Erl+Sor vs. Lin | NA | NA | NA | 0.89 (0.70, 1.12) | Low^4^ | 0.88 (0.45, 1.71) | Low^4^ |
| Erl+Sor vs. Nin | NA | NA | NA | 1.02 (0.69, 1.52) | Low^4^ | 1.02 (0.53, 1.96) | Low^4^ |
| Erl+Sor vs. Ora | NA | NA | NA | 0.62 (0.43, 0.89) | Low^6^ | 0.63 (0.33, 1.18) | Very Low^4,6^ |
| Erl+Sor vs. Pla | NA | NA | NA | 0.67 (0.48, 0.93) | Moderate | 0.68 (0.40, 1.14) | Moderate^4^ |
| Erl+Sor vs. Sor | NA | 0.93 (0.78, 1.11) | Moderate^4^ | NA | NA | 0.93 (0.58, 1.49) | Moderate^4^ |
| Erl+Sor vs. Sun | NA | NA | NA | 0.72 (0.57, 0.90) | Moderate | 0.72 (0.37, 1.40) | Low^4^ |
| Erl+Sor vs. Tig 2mg + Sor | NA | NA | NA | 0.74 (0.45, 1.23) | Low^4^ | 0.75 (0.37, 1.56) | Low^4^ |
| Erl+Sor vs. Tig 6mg + Sor | NA | NA | NA | 1.11 (0.67, 1.82) | Low^4^ | 1.10 (0.54, 2.27) | Low^4^ |
| Erl+Sor vs. Van 100mg | NA | NA | NA | 1.52 (0.71, 3.26) | Very Low^4,6^ | 1.54 (0.67, 3.51) | Very Low^4,6^ |
| Erl+Sor vs. Van 300mg | NA | NA | NA | 1.12 (0.52, 2.40) | Very Low^4,6^ | 1.13 (0.49, 2.60) | Very Low^4,6^ |
| Eve+Sor vs. Len | NA | NA | NA | 1.21 (0.80, 1.83) | Low^4^ | 1.20 (0.58, 2.52) | Low^4^ |
| Eve+Sor vs. Lin | NA | NA | NA | 1.06 (0.69, 1.61) | Low^4^ | 1.05 (0.51, 2.19) | Low^4^ |
| Eve+Sor vs. Nin | NA | NA | NA | 1.22 (0.72, 2.07) | Low^4^ | 1.21 (0.58, 2.52) | Low^4^ |
| Eve+Sor vs. Ora | NA | NA | NA | 0.74 (0.45, 1.23) | Very Low^4,6^ | 0.75 (0.36, 1.53) | Very Low^4,6^ |
| Eve+Sor vs. Pla | NA | NA | NA | 0.80 (0.49, 1.29) | Low^4^ | 0.81 (0.43, 1.48) | Low^4^ |
| Eve+Sor vs. Sor | NA | 1.11 (0.75, 1.64) | Low^1,4^ | NA | NA | 1.10 (0.62, 1.95) | Low^4^ |
| Eve+Sor vs. Sun | NA | NA | NA | 0.86 (0.57, 1.29) | Low^4^ | 0.85 (0.41, 1.76) | Low^4^ |
| Eve+Sor vs. Tig 2mg + Sor | NA | NA | NA | 0.89 (0.48, 1.64) | Low^4^ | 0.89 (0.40, 1.97) | Low^4^ |
| Eve+Sor vs. Tig 6mg + Sor | NA | NA | NA | 1.32 (0.72, 2.42) | Low^4^ | 1.31 (0.59, 2.92) | Low^4^ |
| Eve+Sor vs. Van 100mg | NA | NA | NA | 1.81 (0.78, 4.19) | Very Low^4,6^ | 1.84 (0.74, 4.48) | Very Low^4,6^ |
| Eve+Sor vs. Van 300mg | NA | NA | NA | 1.33 (0.83, 2.16) | Very Low^4,6^ | 1.35 (0.54, 3.27) | Very Low^4,6^ |
| Len vs. Lin | NA | NA | NA | 0.88 (0.71, 1.08) | Low^4^ | 0.87 (0.45, 1.68) | Low^4^ |
| Len vs. Nin | NA | NA | NA | 1.01 (0.69, 1.48) | Low^4^ | 1.01 (0.53, 1.93) | Low^4^ |
| Len vs. Ora | NA | NA | NA | 0.61 (0.43, 0.87) | Low^6^ | 0.63 (0.33, 1.16) | Very Low^4,6^ |
| Len vs. Pla | NA | NA | NA | 0.66 (0.48, 0.90) | Moderate | 0.67 (0.40, 1.12) | Low^4^ |
| Len vs. Sor | NA | 0.92 (0.80, 1.06) | Low^1,4^ | NA | NA | 0.92 (0.58, 1.47) | Low^4^ |
| Len vs. Sun | NA | NA | NA | 0.71 (0.58, 0.86) | Moderate | 0.71 (0.37, 1.38) | Low^4^ |
| Len vs. Tig 2mg + Sor | NA | NA | NA | 0.74 (0.45, 1.20) | Low^4^ | 0.74 (0.36, 1.52) | Low^4^ |
| Len vs. Tig 6mg + Sor | NA | NA | NA | 1.10 (0.67, 1.78) | Low^4^ | 1.09 (0.53, 2.24) | Low^4^ |
| Len vs. Van 100mg | NA | NA | NA | 1.50 (0.71, 3.20) | Very Low^4,6^ | 1.53 (0.67, 3.46) | Very Low^4,6^ |
| Len vs. Van 300mg | NA | NA | NA | 1.11 (0.52, 2.35) | Very Low^4,6^ | 1.12 (0.49, 2.54) | Very Low^4,6^ |
| Lin vs. Nin | NA | NA | NA | 1.15 (0.78, 1.70) | Low^4^ | 1.15 (0.61, 2.23) | Low^4^ |
| Lin vs. Ora | NA | NA | NA | 0.70 (0.49, 1.00) | Very Low^4,6^ | 0.72 (0.38, 1.32) | Very Low^4,6^ |
| Lin vs. Pla | NA | NA | NA | 0.76 (0.55, 1.04) | Low^4^ | 0.77 (0.45, 1.28) | Low^4^ |
| Lin vs. Sor | NA | 1.05 (0.90, 1.23) | Low^1,4^ | NA | NA | 1.05 (0.66, 1.68) | Low^4^ |
| Lin vs. Sun | NA | NA | NA | 0.81 (0.66, 1.00) | Low^4^ | 0.81 (0.42, 1.58) | Low^4^ |
| Lin vs. Tig 2mg + Sor | NA | NA | NA | 0.84 (0.51, 1.38) | Low^4^ | 0.85 (0.42, 1.74) | Low^4^ |
| Lin vs. Tig 6mg + Sor | NA | NA | NA | 1.25 (0.77, 2.04) | Low^4^ | 1.25 (0.62, 2.54) | Low^4^ |
| Lin vs. Van 100mg | NA | NA | NA | 1.72 (0.80, 3.66) | Very Low^4,6^ | 1.75 (0.76, 3.94) | Very Low^4,6^ |
| Lin vs. Van 300mg | NA | NA | NA | 1.26 (0.59, 2.70) | Very Low^4,6^ | 1.29 (0.55, 2.91) | Very Low^4,6^ |
| Nin vs. Ora | NA | NA | NA | 0.61 (0.38, 0.98) | Low^6^ | 0.62 (0.33, 1.14) | Low^6^ |
| Nin vs. Pla | NA | NA | NA | 0.66 (0.42, 1.03) | Low^4^ | 0.67 (0.40, 1.11) | Low^4^ |
| Nin vs. Sor | 0% | 0.91 (0.64, 1.30) | Low^1,4^ | NA | NA | 0.91 (0.58, 1.44) | Low^4^ |
| Nin vs. Sun | NA | NA | NA | 0.70 (0.48, 1.02) | Low^4^ | 0.70 (0.37, 1.33) | Low^4^ |
| Nin vs. Tig 2mg + Sor | NA | NA | NA | 0.73 (0.40, 1.31) | Low^4^ | 0.74 (0.36, 1.50) | Low^4^ |
| Nin vs. Tig 6mg + Sor | NA | NA | NA | 1.08 (0.60, 1.94) | Low^4^ | 1.08 (0.53, 2.21) | Low^4^ |
| Nin vs. Van 100mg | NA | NA | NA | 1.49 (0.65, 3.38) | Very Low^4,6^ | 1.51 (0.66, 3.43) | Very Low^4,6^ |
| Nin vs. Van 300mg | NA | NA | NA | 1.09 (0.48, 2.49) | Very Low^4,6^ | 1.11 (0.48, 2.52) | Very Low^4,6^ |
| Ora vs. Pla | 0% | 1.08 (0.92, 1.27) | Low^1,4^ | NA | NA | 1.07 (0.76, 1.53) | Low^4^ |
| Ora vs. Sor | NA | NA | NA | 1.50 (1.09, 2.07) | Moderate | 1.47 (0.97, 2.26) | Low^4^ |
| Ora vs. Sun | NA | NA | NA | 1.16 (0.82, 1.64) | Very Low^4,6^ | 1.13 (0.61, 2.16) | Very Low^4,6^ |
| Ora vs. Tig 2mg + Sor | NA | NA | NA | 1.20 (0.68, 2.12) | Very Low^4,6^ | 1.19 (0.60, 2.39) | Very Low^4,6^ |
| Ora vs. Tig 6mg + Sor | NA | NA | NA | 1.79 (1.02, 3.14) | Low^6^ | 1.75 (0.89, 3.49) | Very Low^4,6^ |
| Ora vs. Van 100mg | NA | NA | NA | 2.45 (1.21, 4.97) | Moderate | 2.45 (1.18, 5.05) | Moderate |
| Ora vs. Van 300mg | NA | NA | NA | 1.80 (0.89, 3.66) | Low^4^ | 1.79 (0.86, 3.74) | Low^4^ |
| Pla vs. Sor | 64% | 1.39 (1.06, 1.85) | Low^1,2^ | 1.19 (0.50, 2.78) | Moderate^4^ | 1.37 (1.09, 1.75) | High |
| Pla vs. Sun | NA | NA | NA | 1.07 (0.79, 1.46) | Low^4^ | 1.05 (0.64, 1.79) | Low^4^ |
| Pla vs. Tig 2mg + Sor | NA | NA | NA | 1.11 (0.64, 1.92) | Low^4^ | 1.10 (0.62, 2.01) | Low^4^ |
| Pla vs. Tig 6mg + Sor | NA | NA | NA | 1.65 (0.96, 2.84) | Low^4^ | 1.63 (0.91, 2.96) | Low^4^ |
| Pla vs. Van 100mg | NA | 2.27 (1.15, 4.55) | High | NA | NA | 2.27 (1.19, 4.33) | High |
| Pla vs. Van 300mg | NA | 1.67 (0.84, 3.33) | Moderate^4^ | NA | NA | 1.67 (0.86, 3.20) | Moderate^4^ |
| Sor vs. Sun | NA | 0.77 (0.67, 0.88) | Moderate^1^ | NA | NA | 0.77 (0.49, 1.23) | Low^4^ |
| Sor vs. Tig 2mg + Sor | NA | 0.80 (0.50, 1.28) | Low^1,4^ | NA | NA | 0.81 (0.47, 1.39) | Low^4^ |
| Sor vs. Tig 6mg + Sor | NA | 1.19 (0.74, 1.87) | Low^1,4^ | NA | NA | 1.19 (0.69, 2.05) | Low^4^ |
| Sor vs. Van 100mg | NA | NA | NA | 1.63 (0.78, 3.43) | Low^4^ | 1.66 (0.83, 3.29) | Low^4^ |
| Sor vs. Van 300mg | NA | NA | NA | 1.20 (0.88, 1.64) | Low^4^ | 1.22 (0.60, 2.43) | Low^4^ |
| Sun vs. Tig 2mg + Sor | NA | NA | NA | 1.04 (0.64, 1.70) | Low^4^ | 1.05 (0.51, 2.13) | Low^4^ |
| Sun vs. Tig 6mg + Sor | NA | NA | NA | 1.55 (0.95, 2.51) | Low^4^ | 1.54 (0.75, 3.11) | Low^4^ |
| Sun vs. Van 100mg | NA | NA | NA | 2.12 (1.00, 4.51) | Low^6^ | 2.15 (0.93, 4.88) | Very Low^4,6^ |
| Sun vs. Van 300mg | NA | NA | NA | 1.56 (0.73, 3.32) | Very Low^4,6^ | 1.58 (0.68, 3.59) | Very Low^4,6^ |
| Tig 2mg + Sor vs. Tig 6mg + Sor | NA | NA | NA | 1.49 (0.77, 2.88) | Low^4^ | 1.47 (0.93, 2.32) | Low^4^ |
| Tig 2mg + Sor vs. Van 100mg | NA | NA | NA | 2.04 (0.85, 4.91) | Very Low^4,6^ | 2.06 (0.86, 4.91) | Very Low^4,6^ |
| Tig 2mg + Sor vs. Van 300mg | NA | NA | NA | 1.50 (0.62, 3.62) | Very Low^4,6^ | 1.51 (0.61, 3.60) | Very Low^4,6^ |
| Tig 6mg + Sor vs. Van 100mg | NA | NA | NA | 1.37 (0.57, 3.29) | Very Low^4,6^ | 1.39 (0.58, 3.33) | Very Low^4,6^ |
| Tig 6mg + Sor vs. Van 300mg | NA | NA | NA | 1.01 (0.42, 2.42) | Very Low^4,6^ | 1.03 (0.42, 2.44) | Very Low^4,6^ |
| Van 100mg vs. Van 300mg | NA | NA | NA | 0.74 (0.28, 1.95) | Moderate^4^ | 0.73 (0.46, 1.17) | Moderate^4^ |

Note: HR: Hazard Ratio; CI: Confidence Interval; CrI: Credible Interval; NA: Non-applicable. Reasons for downgrading direct evidence (1 to 5), indirect (4, 6) and Mixed estimates(4, 6, 7): 1. Downgraded because of Risk of Bias; 2. Downgraded because of Inconsistency; 3. Downgraded because of Indirectness; 4. Downgraded because of Imprecision; 5. Downgraded because of Publication Bias; 6. Downgraded because of Intransitivity; 7. Downgraded because of Incoherence.
