# Supplementary material for: First-line targ veted therapies of advanced hepatocellular carcinoma: A Bayesian network analysis of randomized controlled trials
Source: PLoS One. 2020 Mar 5;15(3):e0229492. doi: 10.1371/journal.pone.0229492 (PMC7058293; doi:10.1371/journal.pone.0229492)
Supplement: S5 Table — (DOCX) [file pone.0229492.s008.docx]

S23 Table. Direct, Indirect, and NMA Estimates for G3-5AE with the GRADE Assessment.

| Treatment comparison | *I^2^* | Direct estimate;  HR (95% CI) | Quality of evidence | Indirect estimate;  HR (95% CI) | Quality of evidence | Network estimate;  HR (95% CrI) | Quality of evidence |
| --- | --- | --- | --- | --- | --- | --- | --- |
| Dov vs. Bri | NA | NA | NA | 10.35 (4.64, 23.08) | Moderate | 5.72 (0.28, 123.97) | Low^4^ |
| Erl+Sor vs. Bri | NA | NA | NA | 9.65 (5.54, 16.82) | Moderate | 5.35 (0.25, 115.35) | Low^4^ |
| Eve+Sor vs. Bri | NA | NA | NA | 24.62 (8.69, 69.75) | Moderate^1^ | 5.37 (0.26, 111.72) | Low^1,4^ |
| Lin vs. Bri | NA | NA | NA | 13.47 (8.28, 21.90) | Moderate^1^ | 7.44 (0.37, 154.93) | Low^1,4^ |
| Nin vs. Bri | NA | NA | NA | 1.64 (0.66, 4.06) | Low^1,4^ | 0.83 (0.06, 11.06) | Low^1,4^ |
| Pla vs. Bri | NA | 0.34 (0.23, 0.49) | High | 2.22 (1.31, 3.76) | High | 0.60 (0.09, 3.66) | Low^4,7^ |
| Sor vs. Bri | NA | 7.14 (4.76, 10.00) | High | 1.10 (0.64, 1.87) | Moderate^4^ | 3.98 (0.62, 25.71) | Moderate^4^ |
| Van 100mg vs. Bri | NA | NA | NA | 0.12 (0.03, 0.42) | Moderate | 0.19 (0.01, 4.27) | Low^4^ |
| Van 300mg vs. Bri | NA | NA | NA | 0.17 (0.05, 0.64) | Moderate | 0.29 (0.01, 6.58) | Low^4^ |
| Erl+Sor vs. Dov | NA | NA | NA | 0.10 (0.04, 0.22) | Moderate^1^ | 0.94 (0.03, 27.07) | Low^1,4^ |
| Eve+Sor vs. Dov | NA | NA | NA | 0.93 (0.41, 2.12) | Low^1,4^ | 0.93 (0.03, 26.50) | Low^1,4^ |
| Lin vs. Dov | NA | NA | NA | 1.30 (0.60, 2.83) | Low^1,4^ | 1.30 (0.04, 36.79) | Low^1,4^ |
| Nin vs. Dov | NA | NA | NA | 0.16 (0.05, 0.47) | Moderate^1^ | 0.14 (0.01, 2.98) | Low^1,4^ |
| Pla vs. Dov | NA | NA | NA | 0.21 (0.10, 0.48) | Moderate^1^ | 0.10 (0.01, 1.75) | Low^1,4^ |
| Sor vs. Dov | NA | 0.69 (0.34, 1.41) | Low^1,4^ | NA | NA | 0.69 (0.06, 7.67) | Low^1,4^ |
| Van 100mg vs. Dov | NA | NA | NA | 0.07 (0.02, 0.32) | Very low^1,4,6^ | 0.03 (0.00, 1.55) | Very low^1,4,6^ |
| Van 300mg vs. Dov | NA | NA | NA | 0.11 (0.02, 0.48) | Very low^1,4,6^ | 0.05 (0.00, 2.29) | Very low^1,4,6^ |
| Eve+Sor vs. Erl+Sor | NA | NA | NA | 2.55 (0.89, 7.34) | Low^1,4^ | 1.00 (0.03, 28.79) | Low^1,4^ |
| Lin vs. Erl+Sor | NA | NA | NA | 1.40 (0.83, 2.35) | Low^1,4^ | 1.39 (0.05, 38.78) | Low^1,4^ |
| Nin vs. Erl+Sor | NA | NA | NA | 0.17 (0.07, 0.43) | Moderate^1^ | 0.15 (0.01, 3.09) | Low^1,4^ |
| Pla vs. Erl+Sor | NA | NA | NA | 0.23 (0.13, 0.40) | Moderate | 0.11 (0.01, 1.91) | Low^1,4^ |
| Sor vs. Erl+Sor | NA | 0.74 (0.49, 1.12) | Moderate^4^ | NA | NA | 0.74 (0.07, 8.14) | Moderate^4^ |
| Van 100mg vs. Erl+Sor | NA | NA | NA | 0.08 (0.02, 0.30) | Low^1,6^ | 0.04 (0.00, 1.63) | Very low^1,4,6^ |
| Van 300mg vs. Erl+Sor | NA | NA | NA | 0.12 (0.03, 0.46) | Low^1,6^ | 0.05 (0.00, 2.52) | Very low^1,4,6^ |
| Lin vs. Eve+Sor | NA | NA | NA | 0.55 (0.20, 1.52) | Low^1,4^ | 1.38 (0.05, 40.13) | Low^1,4^ |
| Nin vs. Eve+Sor | NA | NA | NA | 0.07 (0.02, 0.19) | Moderate^1^ | 0.15 (0.01, 3.10) | Low^1,4^ |
| Pla vs. Eve+Sor | NA | NA | NA | 0.09 (0.03, 0.26) | Moderate^1^ | 0.11 (0.01, 1.87) | Low^1,4^ |
| Sor vs. Eve+Sor | NA | 0.29 (0.11, 0.77) | Moderate^1^ | NA | NA | 0.74 (0.07, 7.98) | Low^1,4^ |
| Van 100mg vs. Eve+Sor | NA | NA | NA | 0.03 (0.01, 0.15) | Low^1,6^ | 0.04 (0.00, 1.63) | Very low^1,4,6^ |
| Van 300mg vs. Eve+Sor | NA | NA | NA | 0.05 (0.01, 0.23) | Low^1,6^ | 0.05 (0.00, 2.48) | Very low^1,4,6^ |
| Nin vs. Lin | NA | NA | NA | 0.12 (0.05, 0.30) | Moderate^1^ | 0.11 (0.01, 2.25) | Low^1,4^ |
| Pla vs. Lin | NA | NA | NA | 0.17 (0.10, 0.27) | Moderate^1^ | 0.08 (0.00, 1.31) | Low^1,4^ |
| Sor vs. Lin | NA | 0.53 (0.39, 0.73) | Moderate^1^ | NA | NA | 0.53 (0.05, 5.87) | Low^1,4^ |
| Van 100mg vs. Lin | NA | NA | NA | 0.06 (0.02, 0.21) | Low^1,6^ | 0.03 (0.00, 1.13) | Very low^1,4,6^ |
| Van 300mg vs. Lin | NA | NA | NA | 0.08 (0.02, 0.32) | Low^1,6^ | 0.04 (0.00, 1.70) | Very low^1,4,6^ |
| Pla vs. Nin | NA | NA | NA | 1.35 (0.54, 3.35) | Low^1,4^ | 0.72 (0.06, 8.01) | Low^1,4^ |
| Sor vs. Nin | 0% | 4.35 (1.92, 10.00) | Moderate^1^ | NA | NA | 4.82 (0.77, 31.28) | Low^1,4^ |
| Van 100mg vs. Nin | NA | NA | NA | 0.46 (0.10, 2.13) | Very low^1,4,6^ | 0.24 (0.01, 7.85) | Very low^1,4,6^ |
| Van 300mg vs. Nin | NA | NA | NA | 0.68 (0.14, 3.23) | Very low^1,4,6^ | 0.35 (0.01, 12.15) | Very low^1,4,6^ |
| Sor vs. Pla | 60% | 3.22 (2.20, 4.71) | High | NA | NA | 6.63 (1.45, 33.65) | Moderate^4^ |
| Van 100mg vs. Pla | NA | 0.34 (0.10, 1.18) | Moderate^4^ | NA | NA | 0.32 (0.02, 4.32) | Moderate^4^ |
| Van 300mg vs. Pla | NA | 0.50 (0.14, 1.79) | Moderate^4^ | NA | NA | 0.49 (0.03, 6.67) | Moderate^4^ |
| Van 100mg vs. Sor | NA | NA | NA | 0.11 (0.03, 0.38) | Low^1,6^ | 0.05 (0.00, 0.95) | Very low^1,4,6^ |
| Van 300mg vs. Sor | NA | NA | NA | 0.16 (0.04, 0.59) | Low^1,6^ | 0.07 (0.00, 1.44) | Very low^1,4,6^ |
| Van 300mg vs. Van 100mg | NA | NA | NA | 1.47 (0.25, 8.67) | Moderate^4^ | 1.48 (0.10, 21.26) | Moderate^4^ |

Note: HR: Hazard Ratio; CI: Confidence Interval; CrI: Credible Interval; NA: Non-applicable. Reasons for downgrading direct evidence (1 to 5), indirect (4, 6) and Mixed estimates(4, 6, 7): 1. Downgraded because of Risk of Bias; 2. Downgraded because of Inconsistency; 3. Downgraded because of Indirectness; 4. Downgraded because of Imprecision; 5. Downgraded because of Publication Bias; 6. Downgraded because of Intransitivity; 7. Downgraded because of Incoherence.
