# Supplementary figures and images for: First-line targ veted therapies of advanced hepatocellular carcinoma: A Bayesian network analysis of randomized controlled trials
Source: PLoS One. 2020 Mar 5;15(3):e0229492. doi: 10.1371/journal.pone.0229492 (PMC7058293; doi:10.1371/journal.pone.0229492)

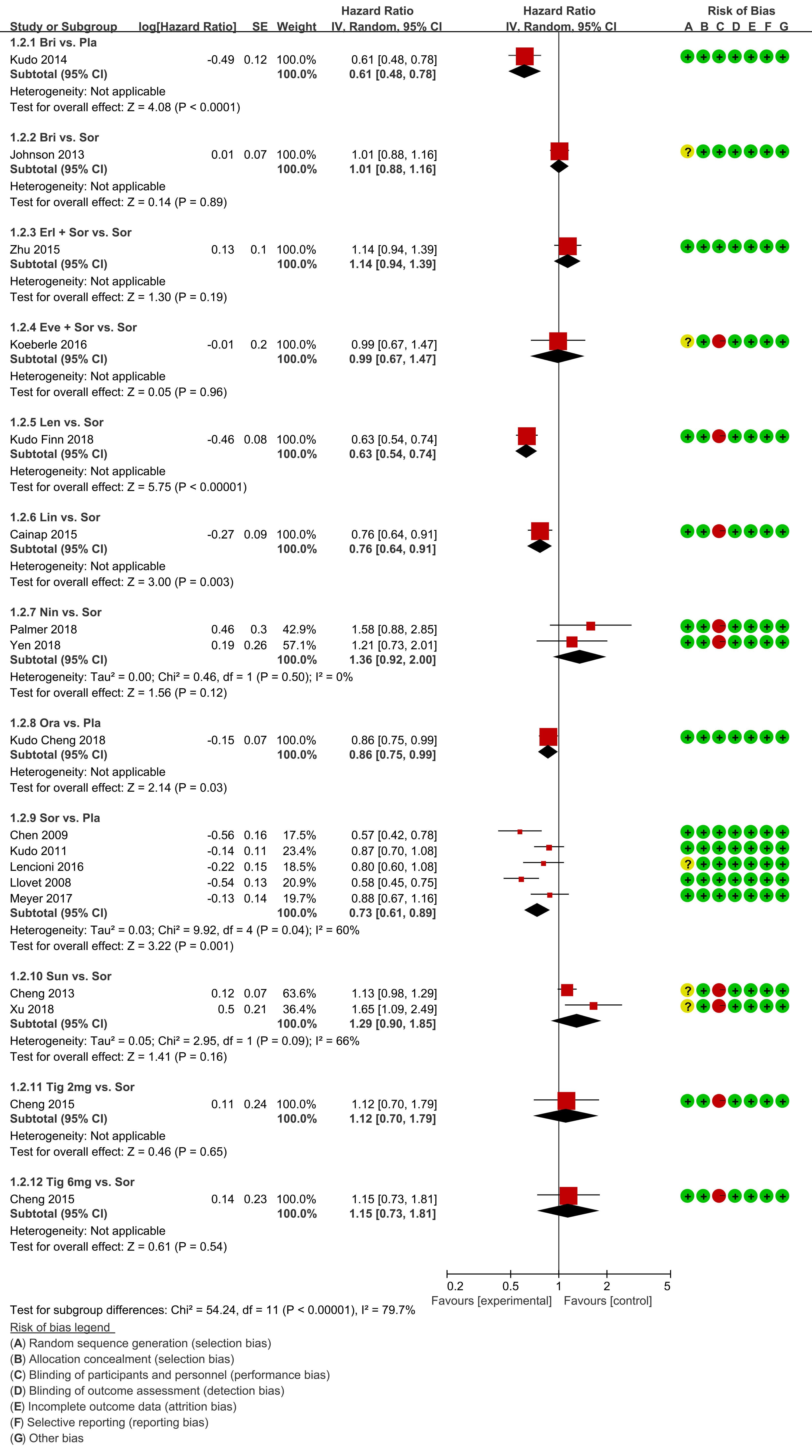

Supplement: S1 Fig — (TIF) [file pone.0229492.s009.tif]

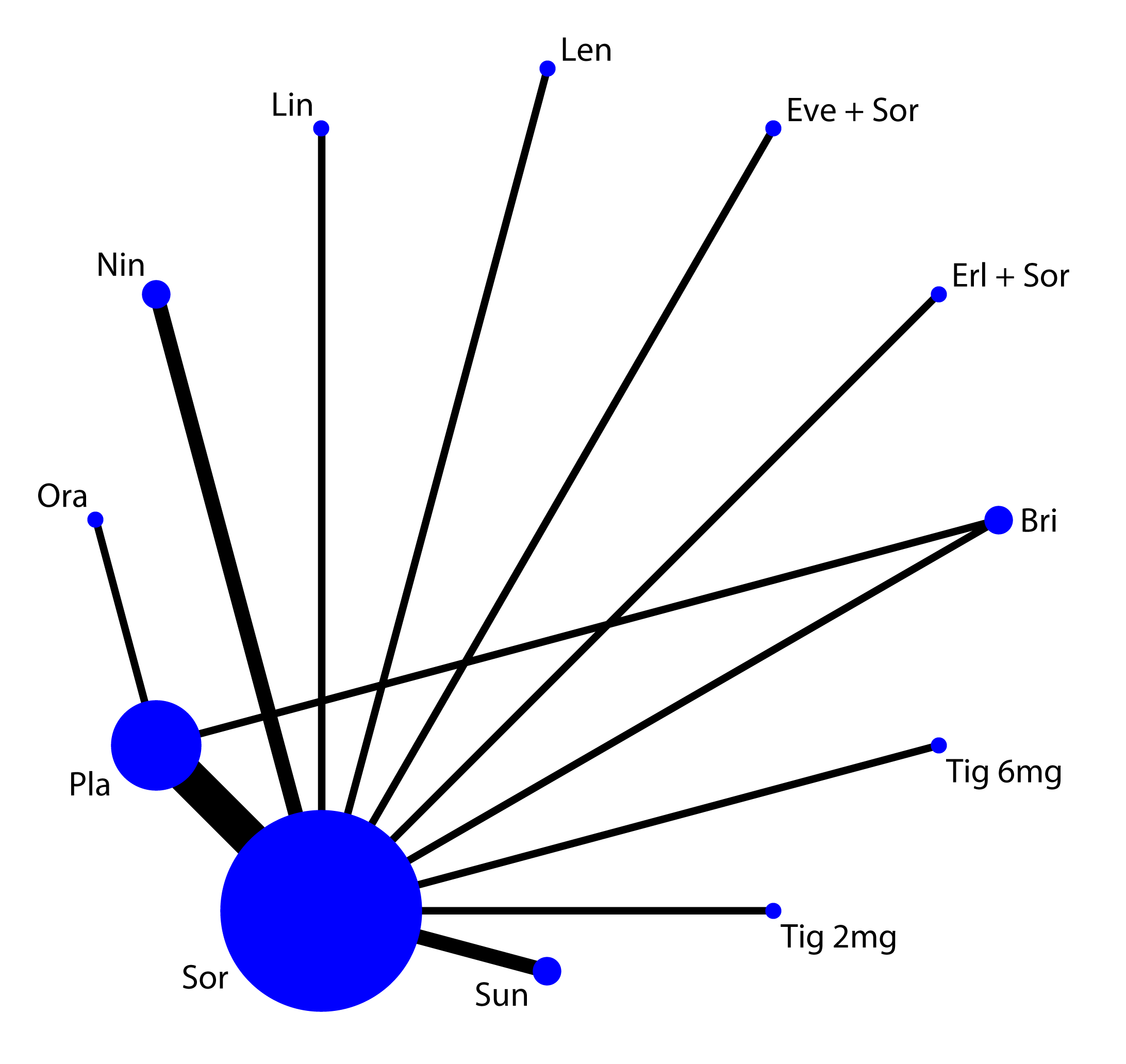

Supplement: S2 Fig — (TIF) [file pone.0229492.s010.tif]

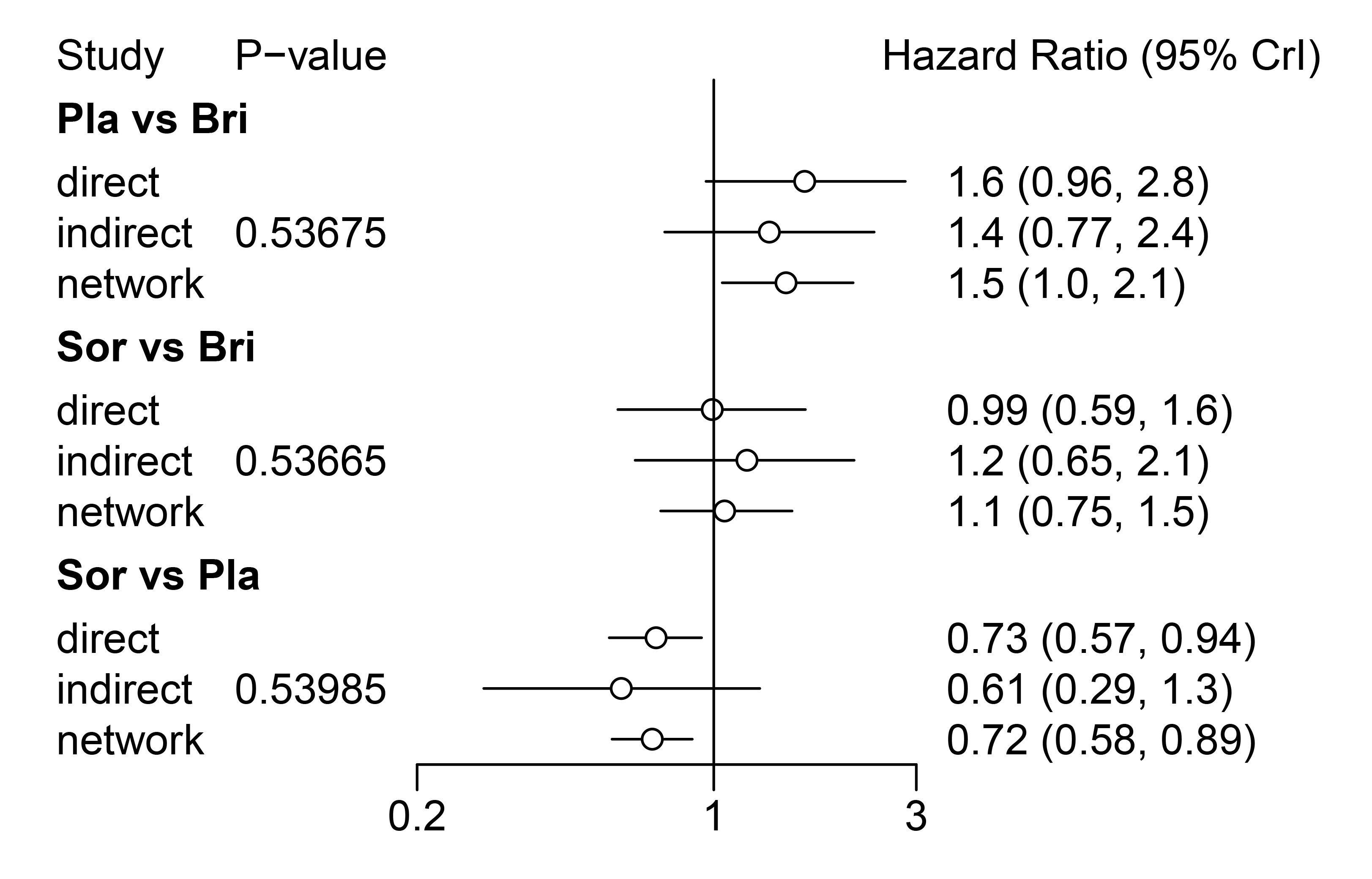

Supplement: S3 Fig — (TIF) [file pone.0229492.s011.tif]

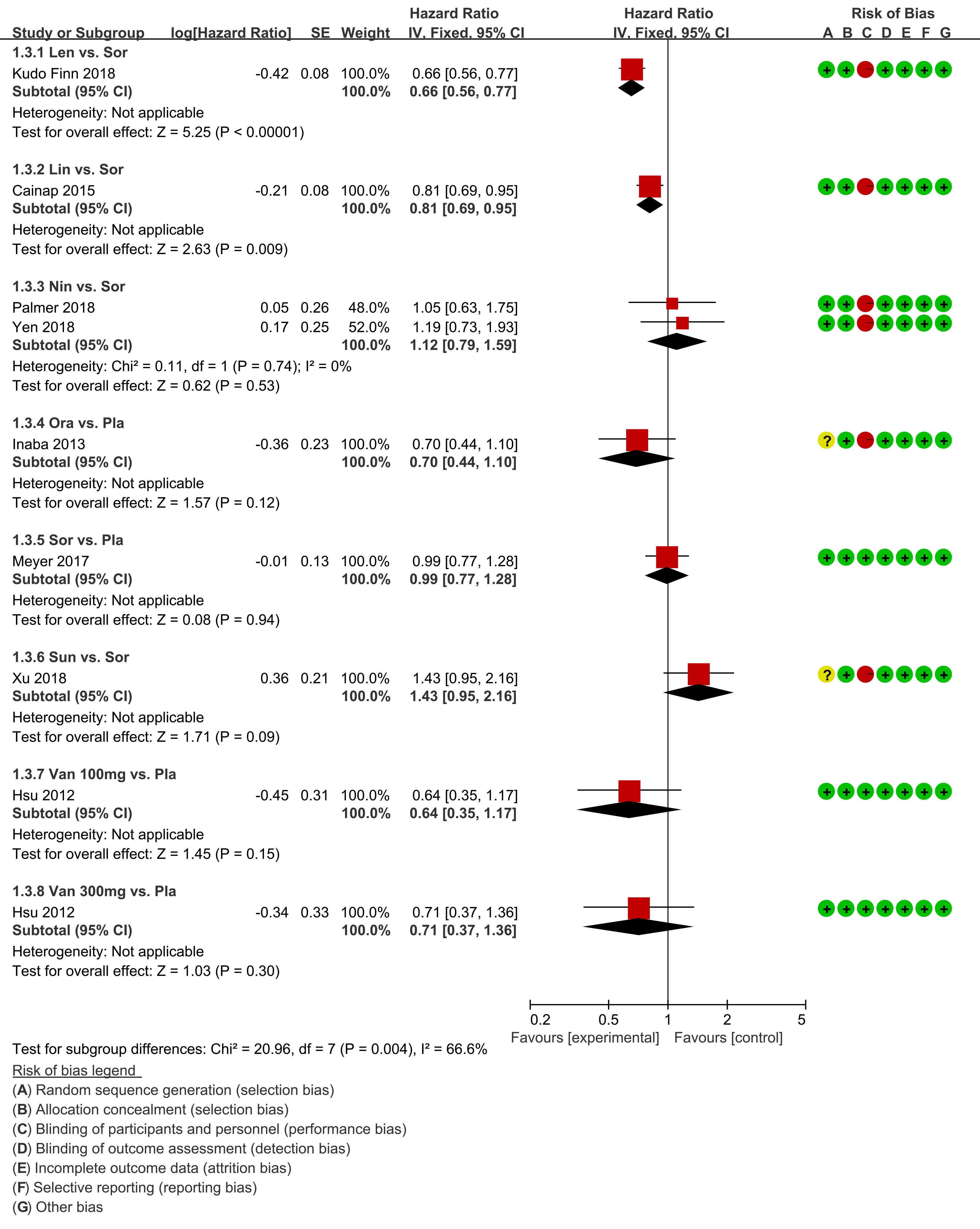

Supplement: S4 Fig — (TIF) [file pone.0229492.s012.tif]

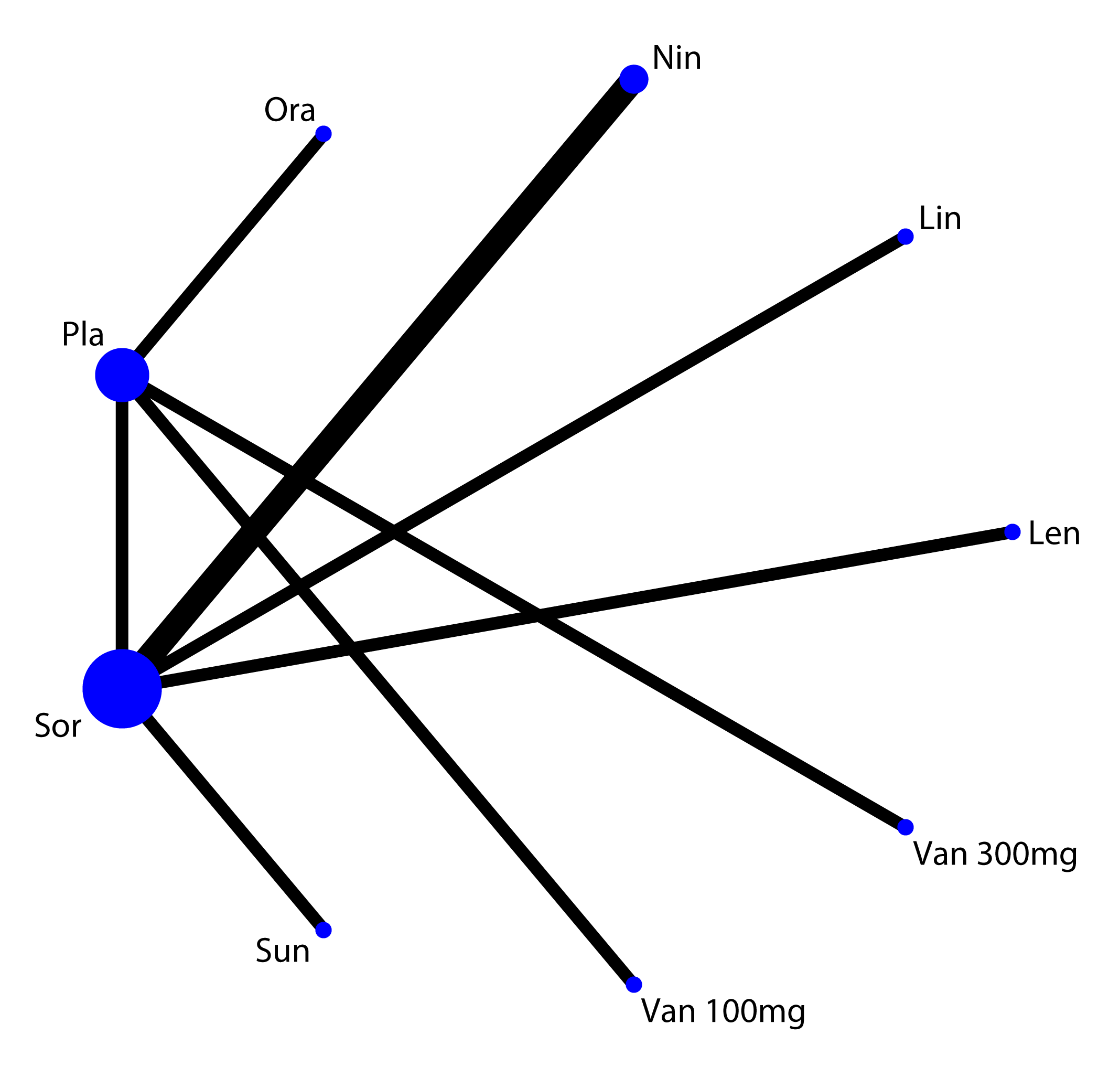

Supplement: S5 Fig — (TIF) [file pone.0229492.s013.tif]

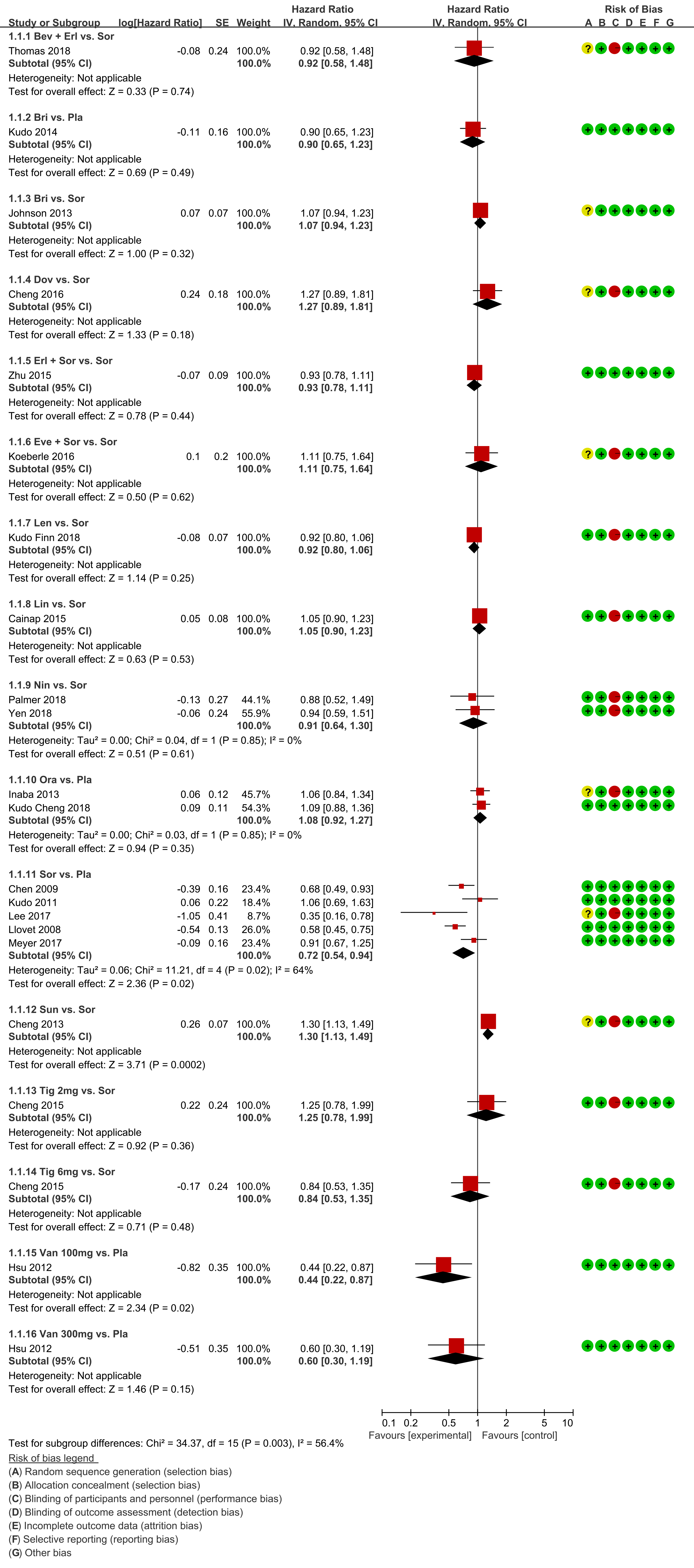

Supplement: S6 Fig — (TIF) [file pone.0229492.s014.tif]

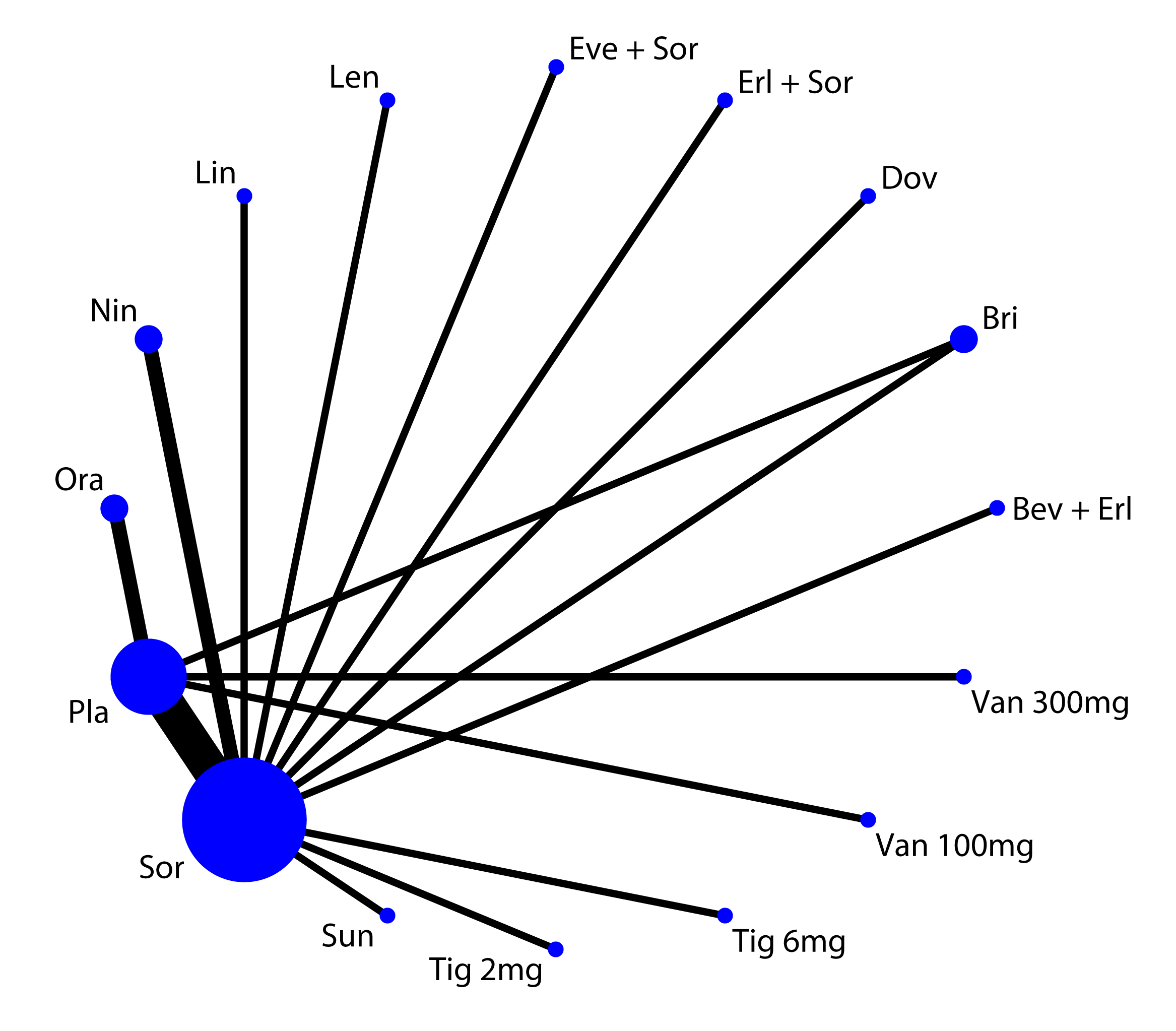

Supplement: S7 Fig — (TIF) [file pone.0229492.s015.tif]

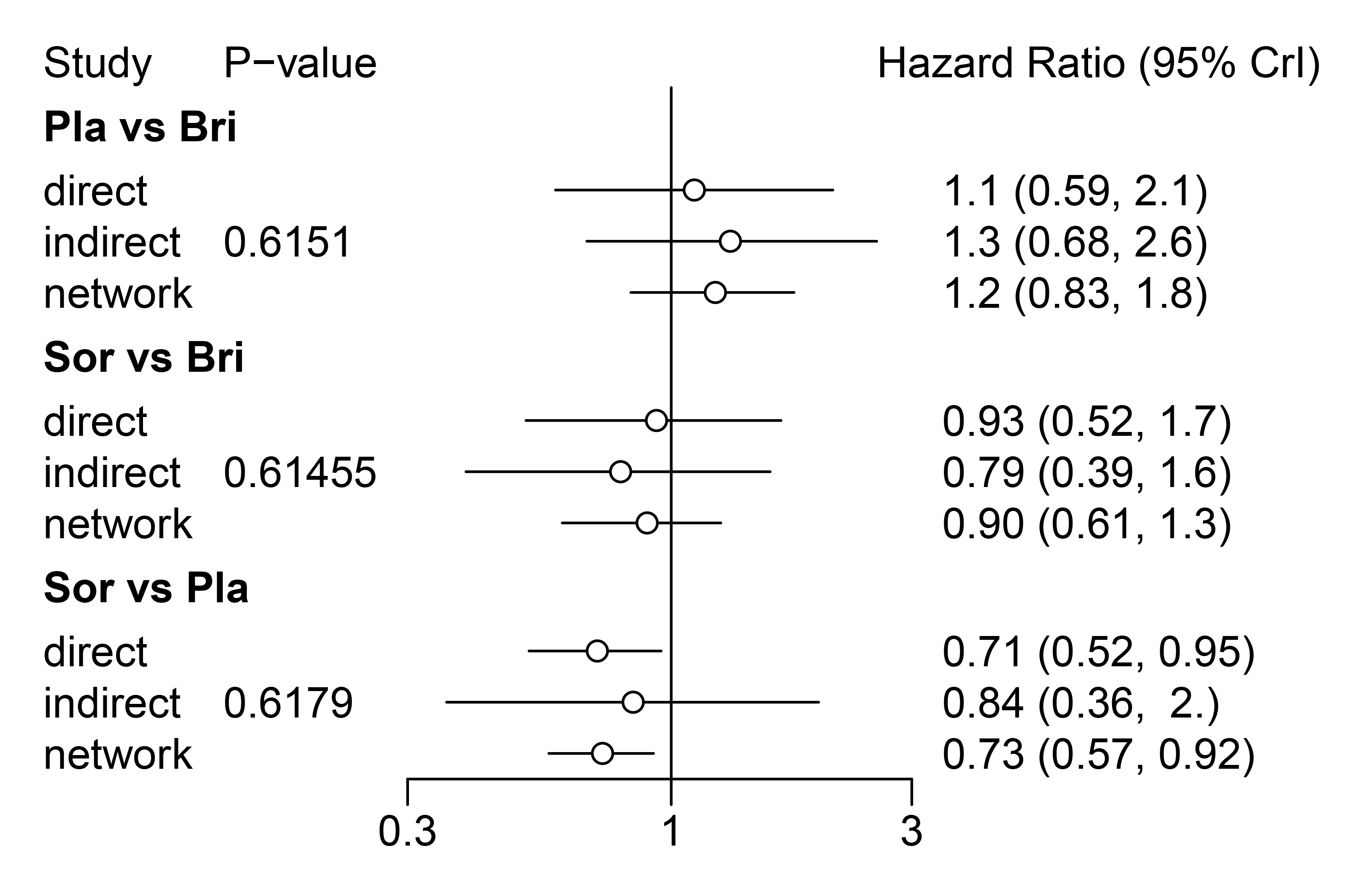

Supplement: S8 Fig — (TIF) [file pone.0229492.s016.tif]

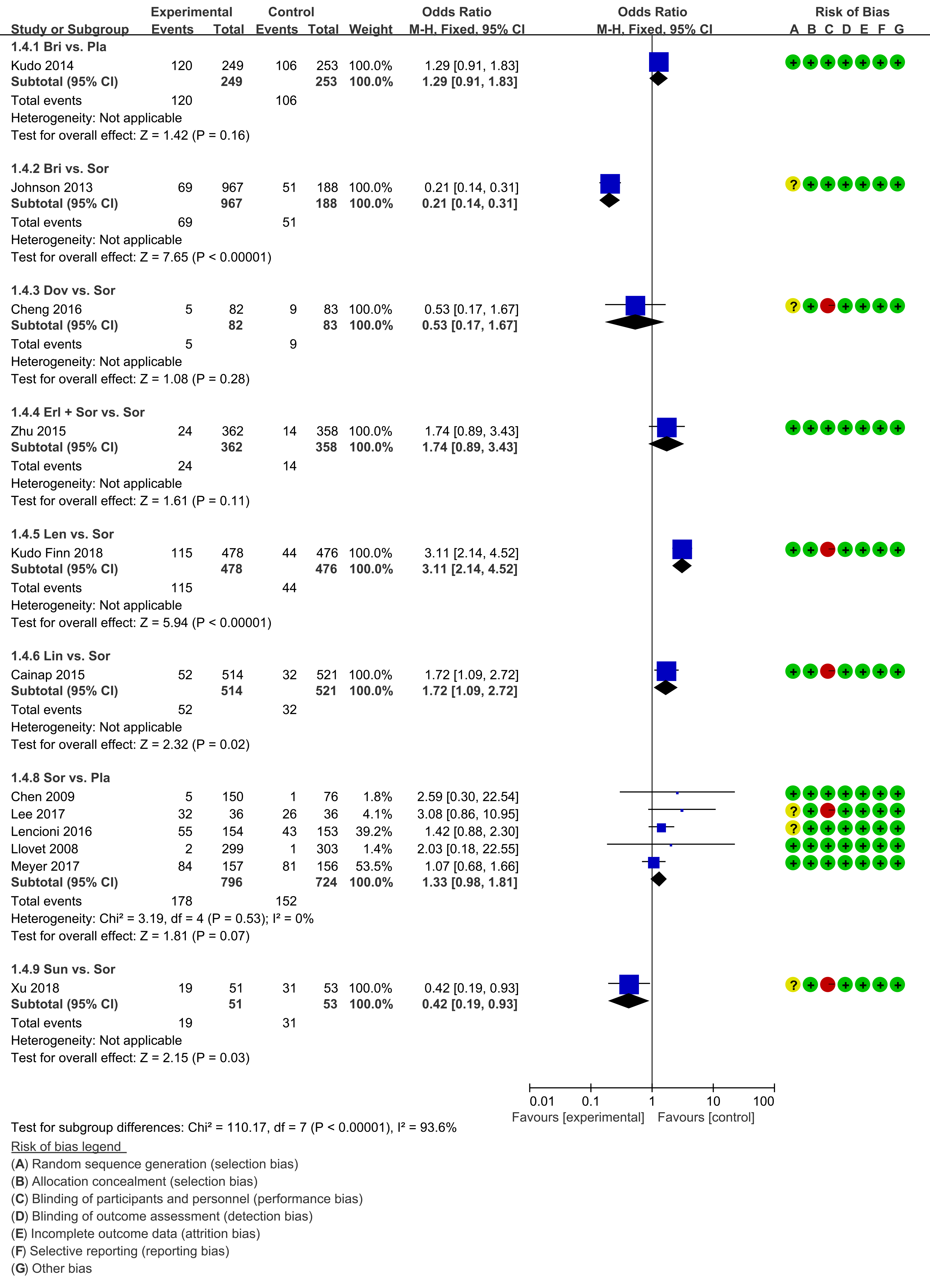

Supplement: S9 Fig — (TIF) [file pone.0229492.s017.tif]

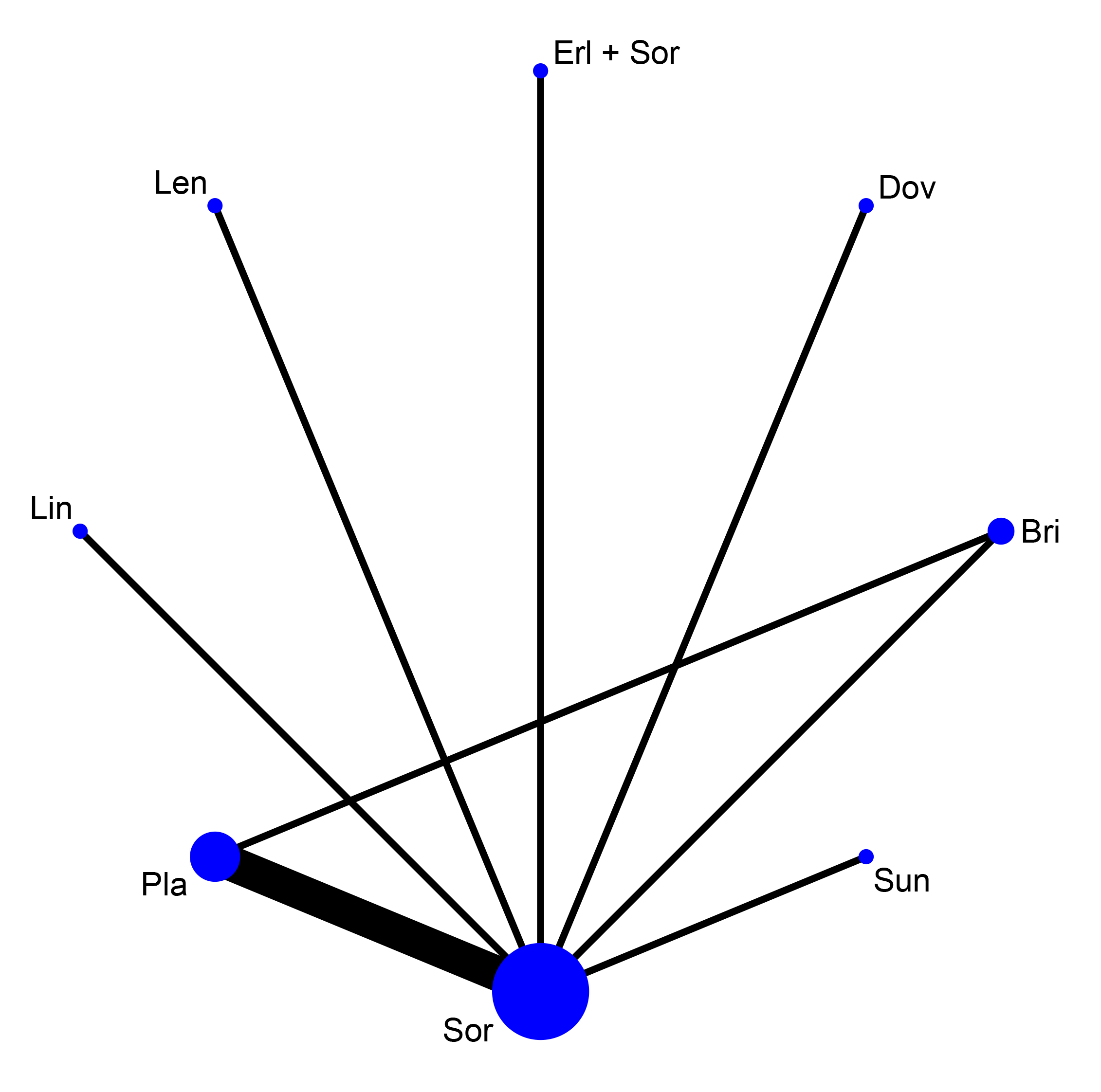

Supplement: S10 Fig — (TIF) [file pone.0229492.s018.tif]

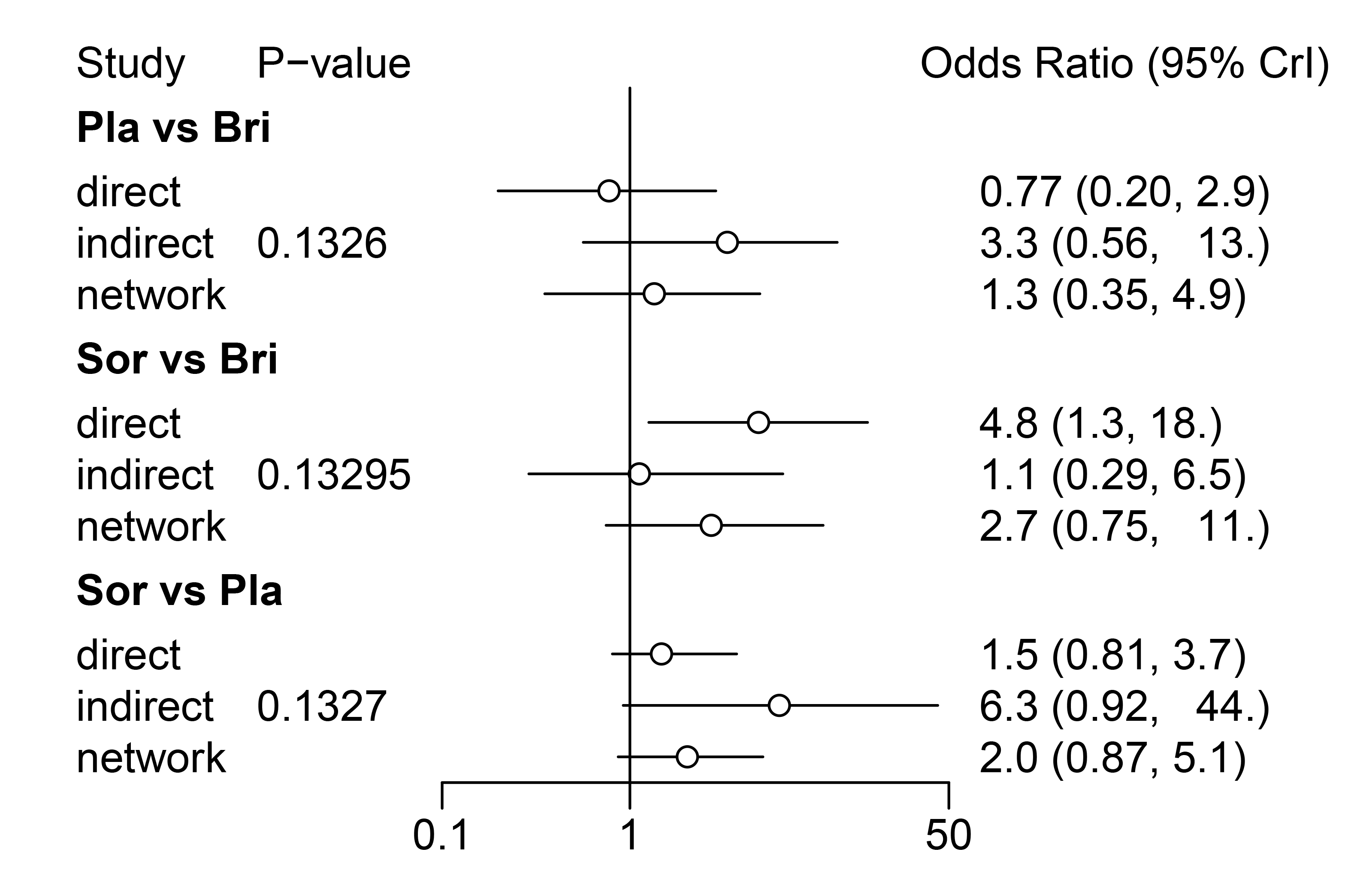

Supplement: S11 Fig — (TIF) [file pone.0229492.s019.tif]

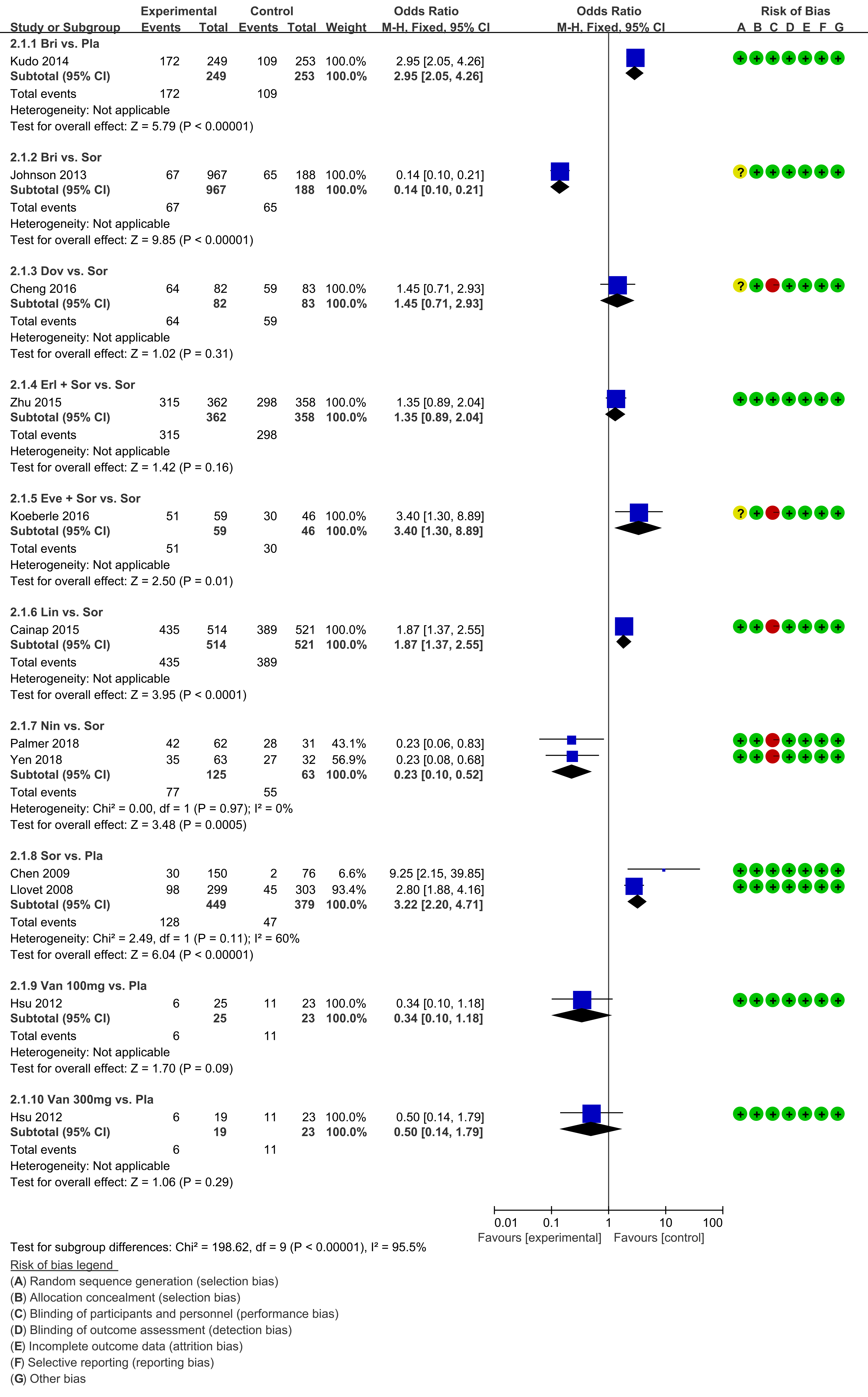

Supplement: S12 Fig — (TIF) [file pone.0229492.s020.tif]

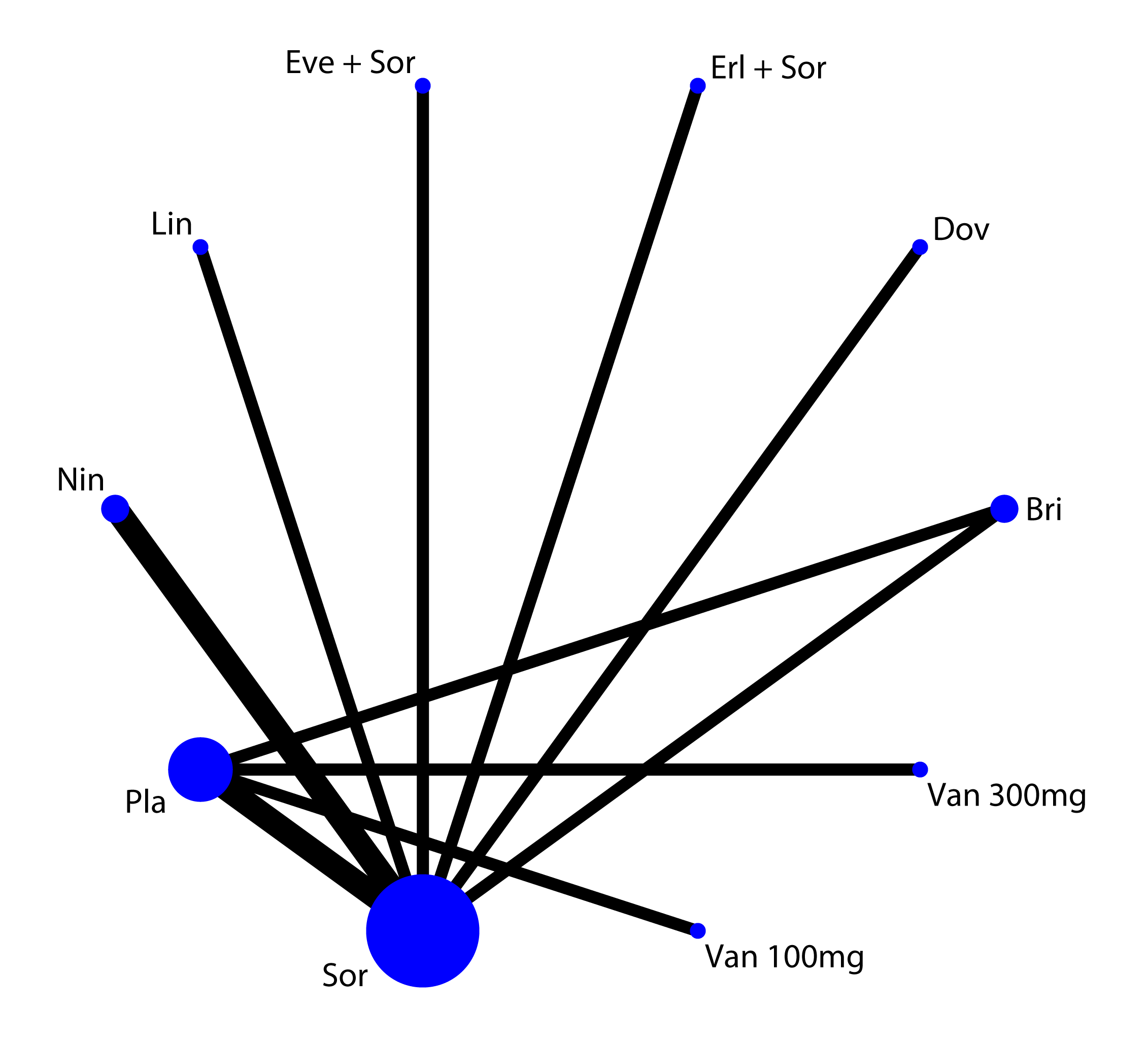

Supplement: S13 Fig — (TIF) [file pone.0229492.s021.tif]

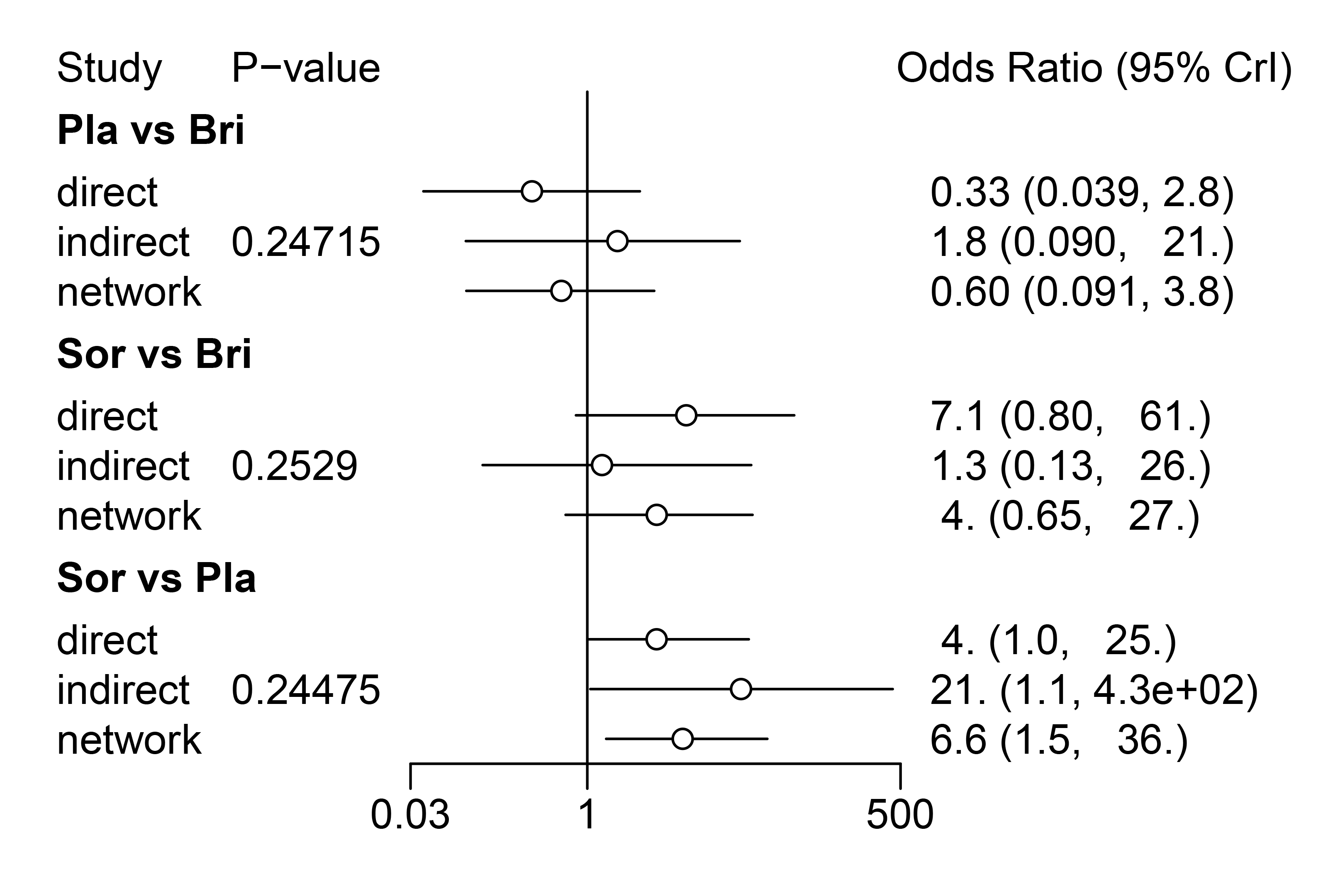

Supplement: S14 Fig — (TIF) [file pone.0229492.s022.tif]

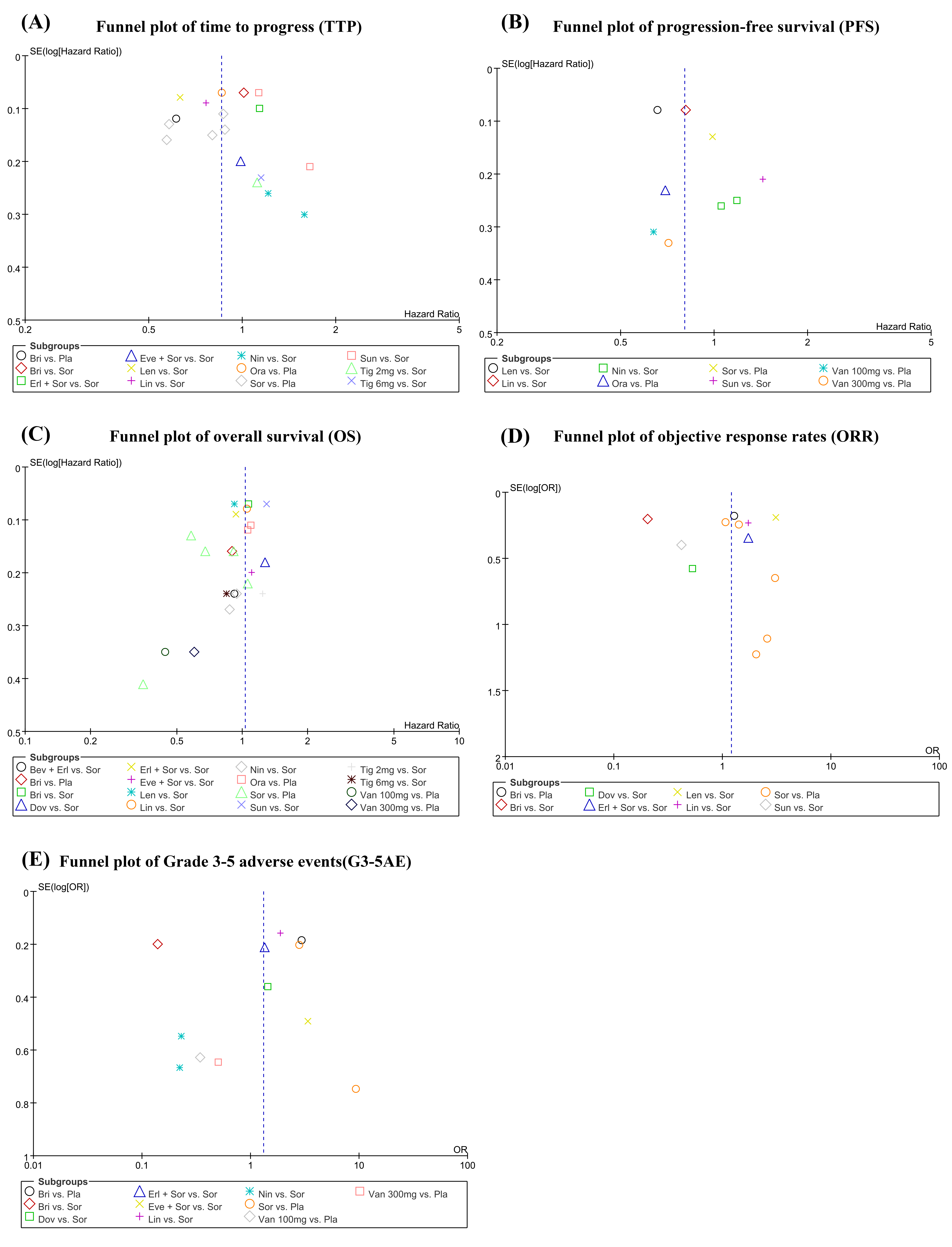

Supplement: S15 Fig — (TIF) [file pone.0229492.s023.tif]

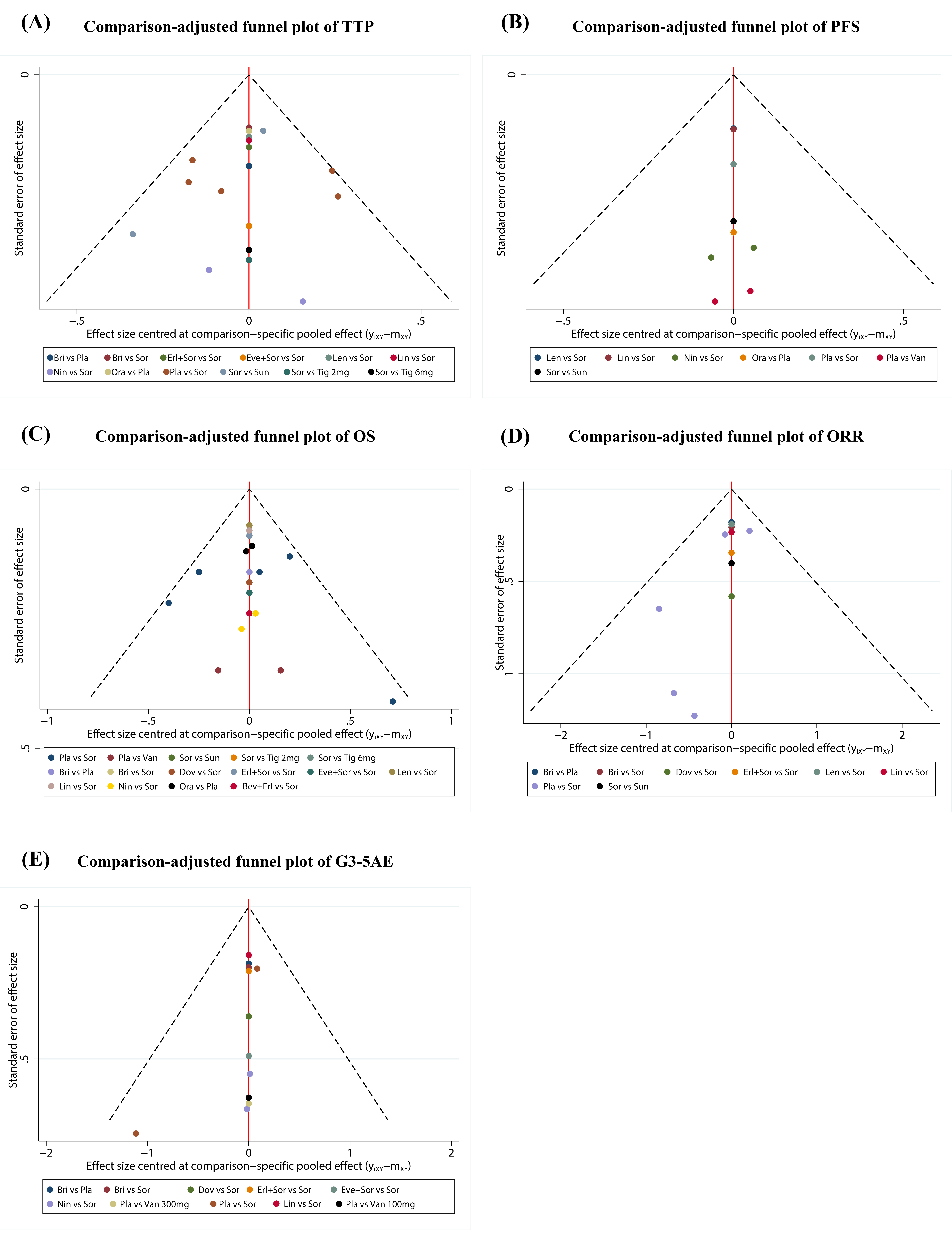

Supplement: S16 Fig — (TIF) [file pone.0229492.s024.tif]
