# Supplementary material for: First-line targ veted therapies of advanced hepatocellular carcinoma: A Bayesian network analysis of randomized controlled trials
Source: PLoS One. 2020 Mar 5;15(3):e0229492. doi: 10.1371/journal.pone.0229492 (PMC7058293; doi:10.1371/journal.pone.0229492)
Supplement: Attachment — Submitted filename: R2R.docx [file pone.0229492.s025.docx]

**First-line targeted therapies of advanced hepatocellular carcinoma: A Bayesian network analysis of randomized controlled trials**

*Replies to Editors*

*1.	QUESTION: Thank you for including your funding statement; "The funders had no role in study design, data collection and analysis, decision to publish, or preparation of the manuscript."*

*ANSWER: The statement "The funders had no role in study design, data collection and analysis, decision to publish, or preparation of the manuscript" has been included within the revised cover letter.*

*2.	QUESTION: Protocol: Reviewer 1 has raised a crucial point regarding the protocol for this nma. One criterion for any systematic review to be of high quality is the presence of a protocol before starting the review. You haven't provided evidence that that protocol existed. At least a register in a database for Systematic reviews should have existed (e.eg., PROSPERO database). Please, either: provide evidence that the protocol/registration existed and was available before stariting the project or explicitly state in the mauscript that the review was not registered in PROSPERO database and did not have an online protocll available, and provide a brief explanation.*

*ANSWER: IT did not have an online protocol available because the protocol was under review in PROSPERO database. We were registered our NMA protocol on July 29, 2019. But the records are being assessed. So, we didn’t have a published protocol.*

*3.	QUESTION: Inconsistency. Reviewer #1 has raised the point on the inconsistency tests. You should explicitly provide the description of the global and the local tests performed to define the inconsistency (per loop), and provide the results of all the tests: the global and each one of the local tests perfomed for each outcome, and for each loop (fo the local). Depending on the tests used: P values, or ROR, should be shown, in the manuscript or in appendices.*

*ANSWER: Modifications were made according to requirements. Consistency checks had been described in detail.*

*4.	QUESTION: Intransitivity: Reviewer #1 has pointed out that you made conclusions on transitivity, that are not supported in the manuscript, to conclude that you need to be clear about how this assumption was evaluated, considering the variables you describe that were used.*

*ANSWER: Modifications were made according to requirements. Intransitivity was also discussed in detail.*

*5.	QUESTION: Certainity of the evidence: Reviewer #1 has recommended to apply GRADE to determine what would be the certainity of the evidence (Also called quality of the evidence) with GRADE. We support that recommendation.*

*ANSWER: Modifications were made according to requirements. We had added the GRADE evaluation system.*

*6.	QUESTION: Heterogeneity: Reviewer #1 pointed out as well that you didn't mention anything on the heterogeneity assessment. Heterogeneity should be evaluated and stataisitically assessed: Both: for the whole netwrok, and for pairwise comparisons. Also, in cases of high heterogeneity, special analyses such as Meta-regression and subgroup analyses shpuld have been performed.*

*ANSWER: Modifications were made according to requirements. Heterogeneity checks had been described in detail.*

*7.	QUESTION: Reviewer #2 has described how your conclusions do not 100% reflect the results.*

*ANSWER: Modifications were made according to requirements. The conclusion had been supplemented by the affirmation of sorafenib.*

*We appreciate for editors/reviewers’ warm work earnestly, and hope that the correction will meet with approval.*

*Thank you and best regards.*

*Yours sincerely,*

*Xuezhong Xu*

*E-mail: xxzdoctor@163.com.*
