# Supplementary material for: First-line targ veted therapies of advanced hepatocellular carcinoma: A Bayesian network analysis of randomized controlled trials
Source: PLoS One. 2020 Mar 5;15(3):e0229492. doi: 10.1371/journal.pone.0229492 (PMC7058293; doi:10.1371/journal.pone.0229492)
Supplement: Attachment — Submitted filename: Response to Reviewers.docx [file pone.0229492.s026.docx]

Dear Editors and Reviewers:

Thank you very much for your comments and suggestions.

We have revised the manuscript, according to the comments and suggestions of reviewers and editor, and responded, point by point to, the comments as listed below.

I would like to re-submit this revised manuscript to **PLOS ONE**, and hope it is acceptable for publication in the journal.

Looking forward to hearing from you soon.

With kindest regards,

Yours Sincerely

Xuezhong Xu.

Replies to Editors

1. **QUESTION:** Reviewer #2 pointed out how the BCLC criteria for the patients could have had an impact on the overall survival. You have pointed out that a subgroup analyses is not possible since the authors didn’t group patients by BCLC criteria. However, there are alternatives to this, considering that you agree that this stage could have an impact on the outcome, and therefore it can be a potential effect modifier.

**-** If authors provided a proportion of patients with a specific BCLC, you could group studies according to specific proportions.

- If a subgroup analyses is not feasible at this point you should address this as a limitation in a paragraph at the end of the discussion section of your manuscript, and discuss there how were the tumor characteristics of the patients and how this may impact on the results.

**ANSWER:** We thank very much for the editor's advice. However, the proportions of patients with different BCLC stages were not uniform. So, the subgroup analysis is not feasible. So, we added some discussion at the end of the discussion section. “The distributions of BCLC stages in different studies were not in full accord. Patients with B or C stage often had worse prognosis than those with A stage. The BCLC criteria for the patients could have an impact on the overall survival. Fortunately, the vast majority of patients include in this analysis were in stage B or C.”

2. **QUESTION:** Regarding reviewer#2 comment about the conclusions, you provide the next sentence: “For the moment, sorafenib was still as a first-line drug of first choice.” We think that according to the results, Srafenib should not be highlighted as the best approach. I think In conclusions you should emphasize in those interventions that were superior, BUT adding the quality of evidence for those. See the following sentence as an example: For OS, Van (100 and 300mg), seem to be the best options with Low and moderate quality of evidence, respectively. For G3 5AE, Van 100 and 300mg), and play seem to be the best interventions, with low quality of evidence all of them (Just as an example, you need to apply GRADE methodology as I suggest below, and find the final quality assessment).

**ANSWER:** We fully endorse the editor's suggestion. We had revised the sentence with “For OS, Van (100 and 300mg), seem to be the best options with low and moderate quality of evidence, respectively. For G3-5AE, Van (100 and 300mg), seem to be the best interventions, with low and very low quality of evidence all of them.”

3. **QUESTION:** Also, regarding this comment from reviewer #2: In the eligibility criteria: 'The key inclusion criteria for study populations: more than 4 weeks since most recent local therapy or no local therapy; no prior systemic therapy.' what does this mean?” You have provided a response, However, it is not reflected in the paper. This part should be clear enough for reader so they could have the same question as Reviewer #2. Thus, please detail in this section, what does that mean and the reasons for that decision.

**ANSWER:** We had revised the sentence with “To avoid the influence of other treatments, the key inclusion criteria for included study populations were as follows: First, it should last more than 4 weeks since most recent local therapy or no local therapy. Second, the patients did not receive prior systemic therapy.”

4. **QUESTION:** - You state that you used the methods recommended by: Puhan et al (2014). However, using only this method is not appropriate as it was the first approach and it has had at least 2 updates: Brignardello-Petersen et al. J Clin Epidemiol 2018, Brignardello-Petersen et al. J Clin Epidemiol 2019, Consider applying the full current approach to assess the quality of evidence.

- According to GRADE approach (Puhan’s and Brignardello-Petersen’s articles) you need to assess the quality of the direct, the indirect and the network evidence for each comparison and each outcome. Although you may present only your Network estimates and your network quality assessments in your main manuscript. However, you need to provide evidence that you conducted all the process appropriately and for that you need to provide all the direct, indirect and NMA estimates + quality of evidence. We cannot identify in your appendices these estimates (Except by some of them that are provided in the Forest plots, and without each GRADE assessment) for readers that would be interested in them.

- Also in page 35 you state:“Therefore, in the present NMA, the GRADE score was downgraded universally due to the diversity of patient characteristics and the disunity of evaluation standard”. This sentence is very problematic, because this is not what GRADE guidance recommends. GRADE working group never recommends to downgrade comparisons “globally”. This shows a misunderstanding of the approach. If you apply GRADE, you should follow all the recommended steps by Puhan’s and Brignardello-Petersen’s articles which are the official GRADE working group Guidance.

- in the same page you state:“Second, it was further downgraded in certain comparisons for no direct evidence”. That may be true. But again, the only way to identify what comparison were downgraded and for what reasons (GRADE Criteria that were use to downgrade in each direct and indirect and NMA estimates)), is to provide in appendices a full list of all the available estimates (D, I, and NMA) for all the outcomes, along with the corresponding GRADE assessment, following the GRADE guidance, and indicating the reasons for downloading each one of them, according to the 7 criteria recommended by the GRADE approach.

- Also, in the same page, you state: “In addition, it was further downgraded in some relevant comparisons because of their IF below 50%”. Could you explain how this criterion match with the GRADE approach recommended by Puhan’s and Brignardello-Petersen 2018 and 2019, One more time, there should be evidence of what comparisons were downgraded by any particular criterion.

- Finally, as you state that you calculated I2. There is no information in the manuscript nor the appendices that shows the i2 for all the direct comparisons. You should provide this data as you described you calculated them to assess the heterogeneity of direct comparisons

**ANSWER:** Thanks very much to the editors for pointing out my shortcomings. We looked through the literature “Brignardello-Petersen R, Bonner A, Alexander PE, Siemieniuk RA, Furukawa TA, Rochwerg B, et al. Advances in the GRADE approach to rate the certainty in estimates from a network meta-analysis. J Clin Epidemiol 2018; 93:36–44. https://doi.org/10.1016/j.jclinepi.2017.10.005 PMID: 29051107”. We have relearned the GRADE approach and rewritten this paragraph. The method was described as follows “For direct comparison, we graded evidence from the five aspects; risk of bias, inconsistency, indirectness, imprecision and publication bias, using the standard GRADE approach. For indirect comparison, we rated evidence according to the lower grades of direct comparisons and intransitivity. For NMA estimates, we rated evidence according to the higher grades of the direct and indirect comparisons and incoherence.” The direct, indirect, and NMA Estimates for OS with the GRADE Assessment were shown in S22-23 Tables. The *I^2^* for all the direct comparisons were shown in S5 Figure, S9 Figure, S11 Figure, S14 Figure and S17 Figure, and they were also shown in S22-23 Tables. We have deleted the “IF” criterion.

5. **QUESTION:** - You excluded one study and the reason provided is that it was data deficient. please clearly explain what does that mean.

- Page 14, line 80, Please write PRISMA in Capital letters.

- Page 14, line 80. There is a specific PRISMA Guidance for NMA, it seems you followed the PRISMA for regular Sr and MA. You need to follow Guidance that is specific for NMA (PRISMA NMA). You also need to provide the appropriate reference for the PRISMA-NMA Guidance.

- Page 14, line 82. Since you cannot provide a PROSPERO registration number at this time, lease indicate the date you submitted the register to the PROSPERO database. Also indicate what was the state of this PROSPERO submission by the time you submitted this manuscript to PlosOne

- FIGURE 4 is of very low quality, you need to provide a better image

- Page 35: They “IF” acronym has not been explained before and has not been detailed in the methods as a key approach

**ANSWER:** - The excluded study was lack of control group.

- Page 14, line 80, we have revised PRISMA in Capital letters. We have rewritten the list following the guidance of PRISMA-NMA checklist.

- Page 14, line 82. This network meta-analysis has been registered in the PROSPERO public database (CRD42019145188)

- FIGURE 4 had been deleted and been replaced by S22-23 Tables.

- Page 35: The “IF” acronym means “information fraction”. It was used to assess statistical power and strength of evidence for each treatment comparison. However, we decide to remove this assessment because the GRADE approach was enough.

Replies to Reviewers

First of all, we thank both reviewers and editors for your positive and constructive comments and suggestions.

**Replies to Reviewer 1:**

1.	**QUESTION:** Based on review comments, authors should have provided a global test for inconsistency assumption, eg. Design-by-treatment interaction model (DBT) and if inconsistency was detected they should have provided a node-splitting approach.

**Answer:** Thanks very much for the advice. We did lack a check for global consistency. However, design-by-treatment interaction model (DBT) was a frequentist NMA model that considers both heterogeneity between studies and inconsistency between study designs according to White IR (White IR. Network meta-analysis. Stata J 2015; 15: 951-985.). Since what I used was bayesian model, design-by-treatment interaction model (DBT) was not suitable for me. In Konstantinos’s study (Konstantinos K, Panagiotis K, Stavros S, et al. Comparative effectiveness of different transarterial embolization therapies alone or in combination with local ablative or adjuvant systemic treatments for unresectable hepatocellular carcinoma: A network meta-analysis of randomized controlled trials[J]. PLOS ONE, 2017, 12(9): e0184597), the author used unrelated mean effects model to evaluate the inconsistency. So, we also used this method to estimate the inconsistency. And the modification has been added in the revised manuscript. The results of comparisons in both consistency and inconsistency models were roughly consistent. The results were shown in S20 Table.

2.	**QUESTION:** I don’t agree with the interpretation provided for the results of the Node-splitting method. Node-splitting approach checks if direct and indirect is in agreement (consistency). Authors concluded to “The result showed a robust and homogeneous network of evidence”. This should be revised. The results of all tests (P-values) for each outcome could be reported in the manuscript with S7, S13, S16 and S19 figures.

**Answer:** The sentence has been revised with “The node-splitting approach also showed a good consistency between the direct and indirect comparisons”. The results of all tests (P-values) for each outcome has been added in the manuscript.

3.	**QUESTION:** All the forest plots provided for NMA should be renamed in figures and tables with Comparison-adjusted funnel plot. Moreover, dashed lines in plots are missing and should be provided (Figure S20).

**Answer:** In S21 Figure, the titles have been revised as Comparison-adjusted funnel plot. And we have added the dashed lines.

We appreciate for editors/reviewers’ warm work earnestly, and hope that the correction will meet with approval.

Thank you and best regards.

Yours sincerely,

Xuezhong Xu

E-mail: xxzdoctor@163.com.
